# Supplementary material for: Requests for futile treatments: what mechanisms play a role? Results of a qualitative study among Dutch physicians
Source: BMJ Open. 2020 Apr 28;10(4):e035675. doi: 10.1136/bmjopen-2019-035675 (PMC7213846; doi:10.1136/bmjopen-2019-035675)
Supplement: Author's manuscript [file bmjopen-2019-035675.draft_revisions.pdf]

BMJ Open is committed to open peer review. As part of this commitment we make the peer review history of every article we publish publicly available.

When an article is published we post the peer reviewers' comments and the authors' responses online. We also post the versions of the paper that were used during peer review. These are the versions that the peer review comments apply to.

The versions of the paper that follow are the versions that were submitted during the peer review process. They are not the versions of record or the final published versions. They should not be cited or distributed as the published version of this manuscript.

BMJ Open is an open access journal and the full, final, typeset and author-corrected version of record of the manuscript is available on our site with no access controls, subscription charges or pay-per-view fees (http://bmjopen.bmj.com).

If you have any questions on BMJ Open's open peer review process please email info.bmjopen@bmj.com

BMJ Open

DRIVERS OF OVERTREATMENT AS DESCRIBED BY VAN DER WAL ACCORDING TO DUTCH PHYSICIANS: RESULTS OF A QUALITATIVE STUDY

Journal:	*BMJ Open*
Manuscript ID	bmjopen-2019-035675
Article Type:	Original research
Date Submitted by the Author:	11-Nov-2019

Complete List of Authors:	van Bruchem-Visser, Rozemarijn Lidewij; Erasmus Medical Center, internal medicine<br>van Dijk, Gert; Erasmus Medical Center, Medical Ethics and Philosophy of Medicine<br>Mattace Raso, Francesco; Erasmus MC, Section of geriatric medicine, department of Internal Medicine<br>de Beaufort, Inez; Erasmus Medical Center, Medical Ethics and Philosophy of Medicine

Keywords:	MEDICAL ETHICS, GENERAL MEDICINE (see Internal Medicine), Quality in health care < HEALTH SERVICES ADMINISTRATION & MANAGEMENT, Health policy < HEALTH SERVICES ADMINISTRATION & MANAGEMENT, QUALITATIVE RESEARCH

SCHOLARONE™ Manuscripts

I, the Submitting Author has the right to grant and does grant on behalf of all authors of the Work (as defined in the below author licence), an exclusive licence and/or a non-exclusive licence for contributions from authors who are: i) UK Crown employees; ii) where BMJ has agreed a CC-BY licence shall apply, and/or iii) in accordance with the terms applicable for US Federal Government officers or employees acting as part of their official duties; on a worldwide, perpetual, irrevocable, royalty-free basis to BMJ Publishing Group Ltd ("BMJ") its licensees and where the relevant Journal is co-owned by BMJ to the co-owners of the Journal, to publish the Work in this journal and any other BMJ products and to exploit all rights, as set out in our licence.

The Submitting Author accepts and understands that any supply made under these terms is made by BMJ to the Submitting Author unless you are acting as an employee on behalf of your employer or a postgraduate student of an affiliated institution which is paying any applicable article publishing charge ("APC") for Open Access articles. Where the Submitting Author wishes to make the Work available on an Open Access basis (and intends to pay the relevant APC), the terms of reuse of such Open Access shall be governed by a Creative Commons licence – details of these licences and which Creative Commons licence will apply to this Work are set out in our licence referred to above.

Other than as permitted in any relevant BMJ Author's Self Archiving Policies, I confirm this Work has not been accepted for publication elsewhere, is not being considered for publication elsewhere and does not duplicate material already published. I confirm all authors consent to publication of this Work and authorise the granting of this licence.

For peer review only

DRIVERS OF OVERTREATMENT AS DESCRIBED BY VAN DER WAL ACCORDING TO DUTCH PHYSICIANS

RESULTS OF A QUALITATIVE STUDY

Corresponding author:

Rozemarijn Lidewij van Bruchem-Visser MD

Postal address: Department of Internal Medicine, Erasmus MC University Medical Center, PO box 2040 3000 CA Rotterdam, The Netherlands

Email: r.l.visser@erasmusmc.nl

Phonenumber: +31107035979

Gert van Dijk

Department of Medical Ethics and Philosophy of Medicine, Erasmus MC University Medical Center, Rotterdam, The Netherlands

g.vandijk.2@erasmusmc.nl

Francesco Mattace-Raso MD PhD

Department of Internal Medicine, Erasmus MC University Medical Center, Rotterdam, The Netherlands

f.mattaceraso@erasmusmc.nl

Inez Duconia de Beaufort PhD

For peer review only - http://bmjopen.bmj.com/site/about/guidelines.xhtml

Department of Medical Ethics and Philosophy of Medicine, Erasmus MC University Medical Center, Rotterdam, The Netherlands

i.debeaufort@erasmusmc.nl

Keywords: medical ethics, general medicine, quality in health care, health policy, qualitative research

Word count: 3440

Abstract

Objectives:

Overtreatment is increasingly seen as a challenge in clinical practice and can lead to unnecessary interventions, poor healthcare outcomes and increasing costs. However, little is known as to what exactly causes overtreatment. In 2015 the Royal Dutch Medical Association addressed this problem and distinguished several mechanisms that were thought to drive overtreatment. In fourteen qualitative interviews among Dutch physicians we investigated which mechanisms played a role in decision-making and whether or not all mechanisms were considered equally important.

Design:

We asked physicians to present a case from personal experience, in which the patient or family requested continuing treatment against the advice of the physician.

Participants:

Fourteen physicians from five different medical area's agreed to participate.

Setting:

Interviews were held face-to-face at the workplace of the physician.

Interventions:

For peer review only

No interventions were used.

Primary and secondary outcome measures:

As this is a qualitative research, no primary and secondary outcome measures were used.

Results:

From these interviews, it was found that three closely related mechanisms were considered to be the main drivers of overtreatment, as perceived by the physician: ‘death is not a common topic of conversation’, ‘never give up’ is the default attitude in our society’ and ‘patients' culture and outlook on life influences their perception of death’.

Conclusions:

Of the fifteen mechanisms described by Van der Wal, not all mechanisms were considered equally important in driving overtreatment. Three mechanisms were mentioned as being the main drivers of overtreatment (‘death is not a common topic of conversation’, ‘never give up’ is the default attitude in our society’ and ‘patients' culture and outlook on life influences their perception of death’), some played no role at all, and others were even considered to be inhibitors of overtreatment, especially the mechanism ‘medical view taking priority’.

For peer review only

For peer review only - http://bmjopen.bmj.com/site/about/guidelines.xhtml

ARTICLE SUMMARY

STRENGTHS AND LIMITATIONS OF THIS STUDY

- Semi-structured interviews in fourteen physicians regarding drivers of overtreatment
- Case-based, no limitations to the type of patient described
- Relatively small group
- Not clear whether the patient or family have the same definition of overtreatment

ACKNOWLEDGEMENTS: We would like to thank all participating physicians

CONFLICT OF INTERESTS: none of the authors have a conflict of interest.

AUTHOR CONTRIBUTIONS:

RvB has made substantial contributions to the conception or design of the work; she has acquired and interpreted the data for the work. She was involved in drafting the work, approved the final version to be published, and agrees to be accountable for all aspects of the work in ensuring that questions related to the accuracy or integrity of any part of the work are appropriately investigated and resolved.

GvD has made substantial contributions to the conception or design of the work. He was involved in drafting the work, approved the final version to be published, and agrees to be accountable for all aspects of the work in ensuring that questions related to the accuracy or integrity of any part of the work are appropriately investigated and resolved.

FMR has made substantial contributions to the conception or design of the work. He was involved in revising the work critically for important intellectual content, approved the final version to be published, and agrees to be accountable for all aspects of the work in ensuring that questions

For peer review only - http://bmjopen.bmj.com/site/about/guidelines.xhtml

related to the accuracy or integrity of any part of the work are appropriately investigated and resolved.

IdB has made substantial contributions to the conception or design of the work. She was involved in revising the work critically for important intellectual content, approved the final version to be published, and agrees to be accountable for all aspects of the work in ensuring that questions related to the accuracy or integrity of any part of the work are appropriately investigated and resolved.

Data statement: Data are stored on a bit locked computer in the Erasmus Medical Center, Rotterdam

Funding: This research received no specific grant from any funding agency in the public, commercial of not-for-profit sectors.

Ethical approval: Ethical approval was not needed for this study. No ethical review board gave ethical approval.

For peer review only

INTRODUCTION

Overtreatment is increasingly seen as a challenge in clinical practice and can lead to unnecessary interventions, poor healthcare outcomes and increasing costs.(1) The occurrence of overtreatment is acknowledged by both patients and physicians in all patients groups, including the elderly.(2-4)

‘Overtreatment’ or ‘too much medicine’ can occur as a result of overdiagnosis, which occurs when people are ‘labeled with or treated for a disease that would never cause them harm’. (5, 6)

Overtreatment is defined in different ways: ‘treatment that is unnecessary or inappropriate’, ‘unnecessary investigations and treatment that lack patient benefit or bear the potential to cause harm’,(7) treatment that is not ‘in line with patient's wishes’, ‘the provision of medical services for which the potential for harm exceeds the potential for benefit’(8) or ‘treatment initiated when there is little or no reliable evidence of a clinically meaningful net benefit, where net benefit equals benefit minus harm’. (9) Overtreatment can concern interventions that can have a positive effect in specific patients , but can harm other patients, for instance percutaneous endoscopic gastrostomy (PEG)-tubes. PEG-tubes can be very beneficiary in some categories of patients, but will harm patients with advanced dementia.(10)

Subjective component

In applying the definition of overtreatment, the patient plays a crucial role. The assessment that a certain intervention is overtreatment in a specific situation can differ between patients, family and physicians. This can make it difficult to objectively determine whether overtreatment has occurred in a specific situation. For instance, what has ‘benefit’ for a patient can often not objectively be determined as patient preferences differ. Some patients will request as much treatment as possible and accept even a small chance of success, while other patients will be much more reluctant in accepting interventions. Medical interventions can also have benefits other than medical. For

For peer review only - http://bmjopen.bmj.com/site/about/guidelines.xhtml

instance: an intervention that has little medical chance of success, can still be seen as 'useful' by the patient, as it can strengthen the feeling that 'something' or 'everything' has been done.

Foster appropriate care

The Royal Dutch Medical Association (RDMA) established a steering group to address the problem of overtreatment and to 'foster appropriate care for those nearing the end of life'. One of the main tasks of the steering group was to identify mechanisms that are thought to drive overtreatment. In 2015, the group published a report 'Just because we can, doesn't mean we should, appropriate end-of-life care.'(11) The report investigated several mechanisms thought to drive overtreatment. These mechanisms play a role in several different domains: society in general, the health care system, industry, professionals, and patients and the public. These mechanisms are in accordance with similar findings from a recent study in Germany.(7) Table 1 shows the fifteen mechanisms that were deemed to be the most important drivers of overtreatment.

Aim of the present study was to investigate which of the mechanisms as described by Van der Wal were recognized by Dutch physicians of different medical specialties to play a role in driving overtreatment. As Van der Wal has primarily focused on patients in the last phase of life, defined as "being of old age or a suffering from a terminal disease with limited life expectancy", we were wondering whether or not these mechanisms indeed play a role in the clinical practice of Dutch physicians.

METHODS

Study setting and population

We purposively sampled eighteen physicians differing in years of working experience and medical specialty to participate. Names were randomly selected from the different departments of (academic) hospitals or groups of local general practitioners. Fourteen physicians from five different medical area's (internal medicine, general practice (GP), intensive care, surgery, and oncology)

For peer review only - http://bmjopen.bmj.com/site/about/guidelines.xhtml

agreed to participate. Their work experience ranged from one to thirty-five years. All interviews were conducted between March 2014 and November 2015.

A medical doctor (RvB, internist geriatrician) conducted semi-structured interviews with all physicians , face-to-face at the workplace of the physician. Before the interview, participants were informed that the interview would deal with “disagreement between physician and patient regarding the course of treatment”. Physicians were asked to think of a case from their own personal experience, in which the patient or family requested continuing of treatment against the advice of the physician. No limitations were given regarding type of underlying disease, patients’ age, sex or cultural background. Participants were given written and verbal information and gave informed consent. The fifteen mechanisms described by Van der Wal (see table 1) were presented and the interviewer asked them whether or not the mechanism played a role in that case.

Data collection and analysis

Interviews were digitally recorded and transcribed verbatim with the interviewees’ permission. Basic demographic information of the described patients (age, sex, ethnicity, religion, relationship with physician) was recorded (see Table 2) as well as of the physicians (sex, medical specialty, years of working experience) (see Table 3). The study was facilitated by QSR NVivo 12 software (QRS International Pty Ltd, Melbourne, Victoria, Australia).

The answers to the mechanisms were coded by two independent researchers (RvB and GvD) to ensure rigorous analysis and to assess whether or not that mechanism was a factor in the described case. Disagreements were settled by consensus. The coding tree was based on the interview guide of the fifteen mechanisms. We used the SRQR checklist when writing our report.(12)

Patient and Public Involvement

Patients or the public were not involved in the design, conduct, or reporting of our research.

For peer review only - http://bmjopen.bmj.com/site/about/guidelines.xhtml

RESULTS

Fourteen different mechanisms were mentioned to play a role in overtreatment, in total 103 times. This could be as a driver of overtreatment (fourteen different mechanisms, in total 86 times, See figure 1), or an inhibitor (four different mechanisms, in total 17 times, See figure 2). Mechanism no. 11 (discussing possible refusal of treatment is more time-consuming) was not mentioned as being a driver or inhibitor of overtreatment. The problem of a lack of time was recognized but in all cases physicians explained they took all the time needed.

We found that according to the interviewed physicians, three closely related mechanisms were considered to be the main drivers of overtreatment: no. 1: 'death is not a common topic of conversation'; no. 2: 'never give up' is the default attitude in our society' and no. 14: 'the great unknown: patients' culture and outlook on life influences their perception of death'. These three mechanisms were mentioned 37 times as being drivers of overtreatment.

Of the mechanisms that were not seen as a driver of overtreatment but rather as a potential inhibitor, mechanism nine: 'medical perspectives often still take priority when it comes to making treatment decisions', was most frequently mentioned as an inhibitor (10 times).

In eleven cases, the physician was not able to communicate directly with the patient, either because the patient spoke a different language and the physician had to communicate by way of the relatives (5 cases) or because the patient was incapacitated (6 cases).

Death is not a common topic of conversation

Closely related with the social tendency to 'not give up' is the fact that patients are often hesitant to discuss the approaching end of life and to accept the fact that they are going to die. This reluctance to talk about the end of life is sometimes enhanced by relatives, who can pressure patients not to give up. This tendency to 'not give up' was considered to be a factor in fifteen out of the 29

For peer review only

described cases. Interestingly, the difficulties of talking about death and end-of-life topics in general, were in most cases attributed to the patient and family.

"The problem was that, he (the husband of the patient) did not want to talk about his wife dying, because that was just not going to happen." (interview 8.2)

"People do not want to think about the end of life, they are still in full fighting mode." (interview 12.1)

It is sometimes thought physicians themselves find it difficult to talk about death, but this is not what we found in our interviews. Physicians described these kind of conversations as being part of their normal daily routine.

"Talking about death is definitely not a problem." (interview 4.1)

"In my line of work, it happens very often that patients die, so it is a logical topic to talk about." (interview 6.1)

However, two physicians described their own struggles with talking about death. In both cases their hope to cure the patient was the cause of reluctance to talk about a bad outcome.

"I found it difficult to talk about, because I was convinced we would start a treatment with intent to cure." (interview 3.1)

"I think it was not discussed before the surgery, that there was also the possibility of Murphy's law with an accumulation of complications. Because you do not want to talk only about all the things that can go wrong." (interview 14.1)

'Never give up' is the default attitude in our society

Dealing with a disease is often seen as a 'fight' or as a 'war'. When dealing with cancer, for instance, patients are supposed to 'fight' the disease. If they are cured they are seen as 'winners' who have

For peer review only

For peer review only - http://bmjopen.bmj.com/site/about/guidelines.xhtml

‘conquered’ the disease. These war-like metaphors are seen also in funding campaigns, such as ‘the war on cancer’. The general tendency in society is therefore ‘to not give up’ in the ‘fight’ against a certain disease. This societal tendency might be seen as one of the drivers of overtreatment. In our interviews, we did indeed find that this mechanism was recognized as being an important factor in almost all cases. In thirteen cases physicians described that the patient or family did not want to give up, even when the physician had told them further treatment would be futile.

“This family, most definitely. They wanted us to pull out all the stops, give all possible treatments.”(interview 10.2)

The tendency to not give up, is not something that always arises from the patient, but can sometimes be pushed by relatives, not from a wish to harm the patient, but out of love and empathy:

“Yes. It does play a role. They would rather have her be subjected to a dozen futile treatments than… (...). They were not trying to make memories with their loved one, no, they were still searching the internet for some treatment the doctor had overlooked.”(interview 12.2)

In cases where there was an acute illness, with little time to decide and often little knowledge about the patient due to the acuteness of the situation, physicians described they automatically opted to start treatment. Situations with easy to cure problems were also described as prompting the physician to not give up.

“When you get a patient with acute renal failure, we have no time to think about other options. To act is the default position.”(interview 6.2)

“I did not want to give up, because I wanted to treat him and keep him from further harm.” (interview 15.1)

For peer review only - http://bmjopen.bmj.com/site/about/guidelines.xhtml

The great unknown: patients' culture and outlook on life influences their perception of death

The cultural and spiritual background of patients can be contributing factors in the perception of physicians that the patient is being subjected to overtreatment. For instance, in some cultures it is not common practice to inform the patients of diagnosis and prognosis. Patients and relatives can also hold specific views on the meaning of suffering and whether or not pain medication is indicated: 'suffering is purification'. These views can bring patients in a situation that physicians find difficult to accept professionally. Physicians described, sometimes in detail, their unfamiliarity with cultural differences between themselves and (family of) their patients.

"On their part, it was most certainly difficult. Because of religion and culture." (interview 5.2)

"We found out too late that this daughter had never told her mother the diagnosis, because in their culture, younger family members were not allowed to convey bad news to their elders." (interview 10.2)

Medical perspectives often still take priority when it comes to making treatment decisions

Van der Wal et al considered one of the drivers of overtreatment to be the idea that physicians take too much of a 'medical' perspective, meaning they focus too much at the medical side of a disease, foregoing other important aspects, such as wellbeing of the patients, or social and cultural aspects. According to Van der Wal this means the 'medical perspective often dominates the decision-making process, even when multidisciplinary consultation (MDO) is involved. Such bodies often consist only of medical specialists, which can overshadow reflection on social, mental, spiritual, cultural and ideological aspects, as well as general well-being'.(11) However, in our interviews, we found this driver needs to be interpreted in a more nuanced way. In our interviews we found physicians may use the medical perspective to convince patients not to continue treatment:

"Well yes. I told them I would not perform a CAT scan because there was no medical reason to do so. So I use the medical perspective to explain why I withhold certain interventions."(interview 7.2)

For peer review only - http://bmjopen.bmj.com/site/about/guidelines.xhtml

"In this case the medical perspective led to our decision to tell the patient 'enough is enough'. Locally, we would be able to do a lot of things, but the fact the same problem would come back in different places, or that the wound would never close, led to us saying 'we will not give any further treatment' ". [interview 10.2]

Taking a medical perspective can therefore mean different things: it can mean the physician takes a perspective in which too much focus is placed on a specific organ, but it can also be an argument not to continue treatment due to medical futility.

For peer review only

For peer review only - http://bmjopen.bmj.com/site/about/guidelines.xhtml

DISCUSSION

In the present study we found that of the fifteen mechanisms described by Van der Wal, not all mechanisms were considered equally important in driving overtreatment. Three mechanisms were mentioned as being the main drivers of overtreatment (‘death is not a common topic of conversation’, ‘never give up’ is the default attitude in our society’ and ‘patients' culture and outlook on life influences their perception of death’), some played no role at all, and others were even considered to be inhibitors of overtreatment, especially the mechanism ‘medical view taking priority’.

These three mechanisms are closely intertwined. For instance, the societal tendency to ‘never give up’ will influence patients and relatives, who might want to continue treatment despite doubts about the effects this will have on the quality or length of live. To talk about death when the patient and family are still aiming for a cure is difficult. Furthermore, in certain cultures it is not customary to discuss the diagnosis, or the approaching death with the patient, making it even harder for the physician to find out what, in a certain case, appropriate care is. As patients and relatives find it difficult to discuss and accept approaching death, physicians often find themselves in a position where patients preferences and values are unknown.

As the interviews were held with physicians, the judgment whether overtreatment played a role in this specific case was seen from a physician’s perspective. It remains unclear whether the patients or relatives also considered the proposed intervention to be a case of overtreatment. This would require further research. The question remains therefore, whether physicians and relatives have similar views on whether or not a certain intervention is to be considered overtreatment

In their report, the committee of Van der Wal investigated these mechanisms in various ways, such as conducting literature studies, consultations with experts from a range of disciplines and independent research. However, the report was a consensus document and a wider evidence base is

For peer review only - http://bmjopen.bmj.com/site/about/guidelines.xhtml

required, especially since the report focused on patients in the last phase of life. It is unknown whether these same mechanisms will apply on other cases of perceived overtreatment.

Interestingly, we found that one of the mechanisms found by Van der Wal et al, the 'medical view taking priority', was not a driver of overtreatment according to the interviewed physicians, but instead was often used as an inhibitor. In our findings the 'medical view', was used by physicians as an argument to refuse to provide the intervention under discussion, as the intervention was deemed medically futile by the physician. In the view of Van der Wal et al, physicians will sometimes focus too much on a certain organ – i.e. the heart or kidneys – and fail to see the 'whole' of the patient. However, this was not what we found. Of course this might be due to the fact we interviewed physicians, who might have difficulty recognizing this mechanism as playing a role in their relationship with a patient. Another important finding was that, when asked for a case from their own experience, physicians often presented cases in which the patient was not able to communicate with the physician by himself, either due to a language barrier of mental incapacity. In these cases it was therefore the relatives and not the patient that opposed the advice of the physicians. In the presented cases relatives play a central role in the wish to continue treatment against the advice of the physician. The fact that the wishes of the patient were in all cases unknown to the physician and the relatives enhanced the problem.

One of the reasons for the fact that physicians presented a case in which the relatives played a crucial role might be the subjective aspect of overtreatment. As overtreatment is, at least partly, a subjective phenomenon, physicians might not consider an intervention to be overtreatment when it is the patient him or herself that asks for a specific intervention, as the patient has consented or has specifically asked for the intervention. Patients can have different views on what is appropriate care towards the end of life, and physicians are likely to be willing to go along with the patient – within limits, as the physicians will not likely go along with wishes of the patients that are deemed

For peer review only - http://bmjopen.bmj.com/site/about/guidelines.xhtml

medically futile - as long as the patient is well informed and is aware of the risks and possible benefits (or lack thereof) of the intervention.

However, when it is not the patient but relatives that go against the advice of the physician, physicians might sooner consider the intervention to be overtreatment, as they feel the intervention does not serve the medical interest of the patient, the patient has not consented to the procedure, and might actually suffer from the procedure. A refusal of pain medication or sedatives, or a request for a certain intervention will probably be judged in a different manner by the physician if it comes from the patient as opposed to a request from relatives. Although relatives will almost certainly have the best intentions – 'do not let my loved one die' – these intentions might have negative consequences for the patient. It might be acceptable for a physician to see a patient suffer when that patient has deliberately accepted negative consequences of a certain intervention. However, in a mentally incapacitated patient, suffering because of medically futile treatments of any kind is more difficult to accept.

The important role of relatives is not mentioned by Van der Wal as a distinct mechanism that drives overtreatment. This could be explained by the fact that the role of relatives was not a subject of their research. We suggest however, that future research should focus more on the role of relatives in the debate on overtreatment, and the reasons they have for continuation or start of treatments that are deemed inappropriate by the physician. For instance, it would be of interest to find out whether the relatives in these situations considered the intervention to be a case of overtreatment as well, or whether they considered the situation to be appropriate care.

For peer review only

Conclusions

We have found that of the fifteen mechanisms as described by Van der Wal, three contributing mechanisms were identified as the major driving factors in overtreatment: 'death is not a common topic of conversation', 'never give up' is the default attitude in our society' and 'the great unknown: patients' culture and outlook on life influences their perception of death'.

The mechanism 'medical perspectives often still take priority when it comes to making treatment decisions' was mentioned as being an inhibitor of overtreatment in the majority of interviews. In many cases the relatives play a crucial role in the wish to continue treatment against the advice of the physician. Overtreatment was defined from the perspective of the physicians. Further research is needed to investigate whether relatives of the patient define the same situations as 'overtreatment'.

The findings from this study underline the importance of advance care planning and a timely discussion on patients wishes and preferences regarding the end of life, so as to avoid overtreatment and to foster appropriate care.

For peer review only

Table 1: Mechanisms described bij Van der Wal et al

1	Death is not a common topic of conversation
2	‘Never give up’ is the default attitude in our society
3	Action is better than inaction
4	Professional guidelines focus on ‘action’
5	Education focuses on ‘action’
6	Physicians are payed for treatment
7	With so many care providers and so little coordination, who is responsible?
8	No holistic view of the patient
9	Medical perspectives often still take priority when it comes to making treatment decisions.
10	Palliative care is initiated too late
11	Discussing possible refusal of treatment is more time-consuming
12	To talk about death is difficult
13	Uncertainty about what to tell patients
14	The great unknown: patients' culture and outlook on life influences their perception of death
15	People document their wishes and preferences regarding end-of-life care too late, and often not (thorough enough)

For peer review only - http://bmjopen.bmj.com/site/about/guidelines.xhtml

Table 2. Descriptive characteristics of patients

Total number of patients	14
Age	
Aged 25-45	6
Aged 46-65	3
Aged 65 years or older	5
Sex	
Male	4
Female	10
Underlying disease	
Malignancy	5
Kidney disease	2
Diabetes mellitus	0
Neurological condition	4
Chronic Obstructive Pulmonary Disease	0
Infection	0
Complex surgery	1
Medically unexplained	1
Dementia	1
Religious background	
Non-religious/not mentioned	4
Muslim	5
Christian	2
Other	3
Existing relationship patient-physician	7
Mentally competent	8
Patient communicator	5
Family communicator	3
Mentally incompetent	6

For peer review only - http://bmjopen.bmj.com/site/about/guidelines.xhtml

Table 3. Descriptive characteristics of the interviewed physicians

Participant	Sex	Specialty	Working experience (years)
1	Female	General practitioner	22
2	Male	Intensive Care	10
3	Female	Oncology	11
4	Male	Surgery	30
5	Female	Intensive Care	35
6	Male	Internal Medicine	4
7	Female	Oncology	20
8	Female	General practitioner	11
9	Female	Internal Medicine	1
10	Female	Internal Medicine	15
11	Male	Surgery	1
12	Male	Surgery	10
13	Female	Oncology	2
14	Female	Intensive Care	6
15	Female	General practitioner	3

For peer review only

For peer review only - http://bmjopen.bmj.com/site/about/guidelines.xhtml

Figure 1. Drivers of overtreatment, attributed to different parties

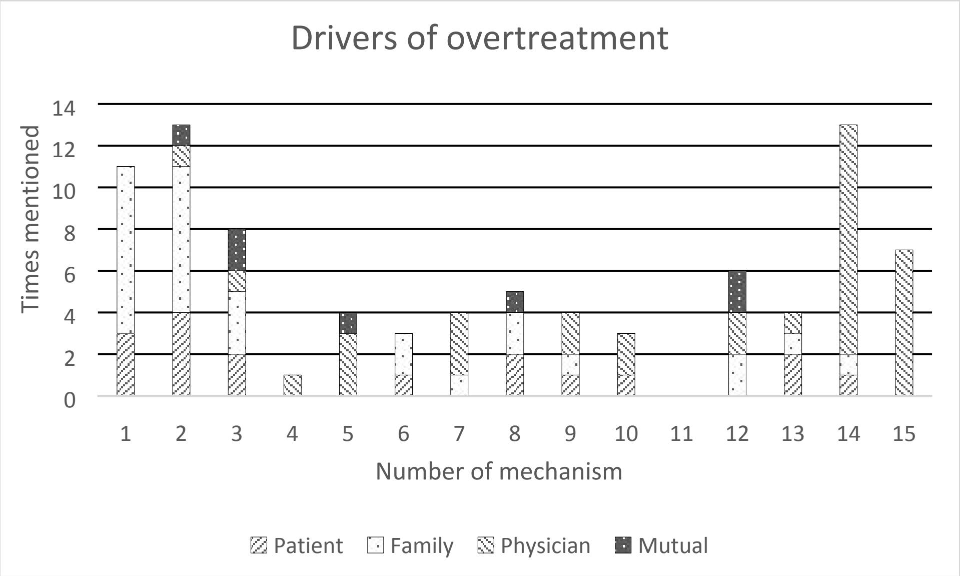

For peer review only

Figure 2. Inhibitors of overtreatment, attributed to different parties

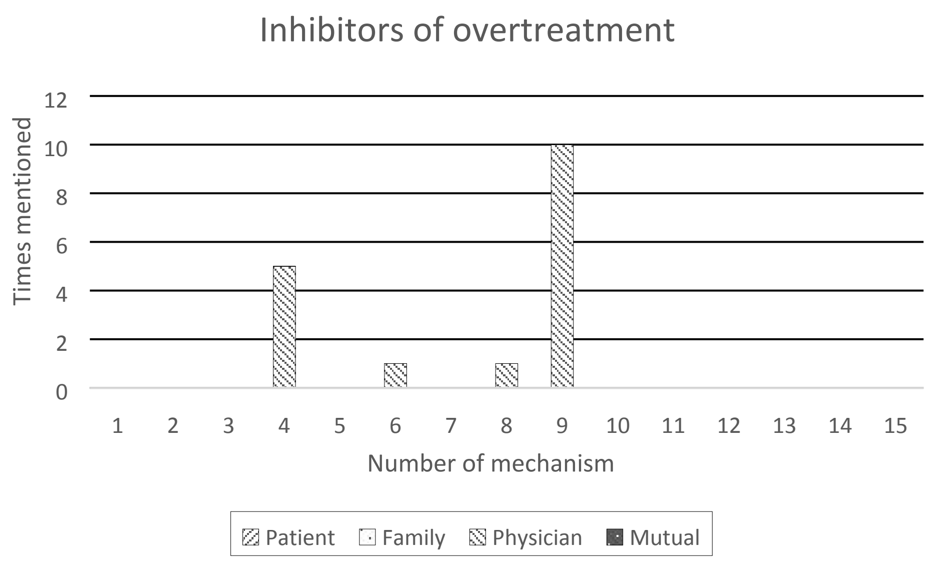

For peer review only

References

1. Morgan DJ, Wright SM, Dhruva S. Update on medical overuse. JAMA Internal Medicine. 2015;175(1):120-4.
2. How S SA, Lau J, Schoen C. Public Views on US Health System Organization: A Call for New Directions The Commonwealth Fund. 2008(August).
3. Lyu H, Xu T, Brotman D, Mayer-Blackwell B, Cooper M, Daniel M, et al. Overtreatment in the United States. PLoS One. 2017;12(9):e0181970.
4. Thorsteinsdottir B, Swetz KM, Tilburt JC. Dialysis in the frail elderly--a current ethical problem, an impending ethical crisis. J Gen Intern Med. 2013;28(11):1511-6.
5. Morgan DJ, Leppin AL, Smith CD, Korenstein D. A Practical Framework for Understanding and Reducing Medical Overuse: Conceptualizing Overuse Through the Patient-Clinician Interaction. J Hosp Med. 2017;12(5):346-51.
6. Pathirana T, Clark J, Moynihan R. Mapping the drivers of overdiagnosis to potential solutions. BMJ. 2017;358:j3879.
7. Alber K, Kuehlein T, Schedlbauer A, Schaffer S. Medical overuse and quaternary prevention in primary care - A qualitative study with general practitioners. BMC Fam Pract. 2017;18(1):99.
8. Chassin MR, Galvin RW. The urgent need to improve health care quality. Institute of Medicine National Roundtable on Health Care Quality. JAMA. 1998;280(11):1000-5.
9. Herzstein J, Ebell M. Improving quality by doing less: overtreatment. Am Fam Physician. 2015;91(5):289-91.
10. van Bruchem-Visser RL, Mattace-Raso FUS, de Beaufort ID, Kuipers EJ. Percutaneous endoscopic gastrostomy in older patients with and without dementia: Survival and ethical considerations. J Gastroenterol Hepatol. 2019;34(4):736-41.
11. Steering Committee for Appropriate End-of-Life Care. Just because we can, doesn't mean we should. 2015.
12. O'Brien BC, Harris IB, Beckman TJ, Reed DA, Cook DA. Standards for reporting qualitative research: a synthesis of recommendations. Academic Medicine. 2014;89(9):1245-51.

For peer review only

For peer review only - http://bmjopen.bmj.com/site/about/guidelines.xhtml

Reporting checklist for qualitative study.

Based on the SRQR guidelines.

Instructions to authors

Complete this checklist by entering the page numbers from your manuscript where readers will find each of the items listed below.

Your article may not currently address all the items on the checklist. Please modify your text to include the missing information. If you are certain that an item does not apply, please write "n/a" and provide a short explanation.

Upload your completed checklist as an extra file when you submit to a journal.

In your methods section, say that you used the SRQRreporting guidelines, and cite them as:

O'Brien BC, Harris IB, Beckman TJ, Reed DA, Cook DA. Standards for reporting qualitative research: a synthesis of recommendations. Acad Med. 2014;89(9):1245-1251.

		Reporting Item	Page Number
**Title**			
	#1	Concise description of the nature and topic of the study identifying the study as qualitative or indicating the approach (e.g. ethnography, grounded theory) or data collection methods (e.g. interview, focus group) is recommended	1
**Abstract**			
	#2	Summary of the key elements of the study using the abstract format of the intended publication; typically includes background, purpose, methods, results and conclusions	2-3
**Introduction**			
Problem formulation	#3	Description and signifcance of the problem / phenomenon studied: review of relevant theory and empirical work; problem statement	6-7
Purpose or research question	#4	Purpose of the study and specific objectives or questions	7

For peer review only

Methods

Qualitative approach and research paradigm	#5	Qualitative approach (e.g. ethnography, grounded theory, case study, phenomenolgy, narrative research) and guiding theory if appropriate; identifying the research paradigm (e.g. postpositivist, constructivist / interpretivist) is also recommended; rationale. The rationale should briefly discuss the justification for choosing that theory, approach, method or technique rather than other options available; the assumptions and limitations implicit in those choices and how those choices influence study conclusions and transferability. As appropriate the rationale for several items might be discussed together.	8
Researcher characteristics and reflexivity	#6	Researchers' characteristics that may influence the research, including personal attributes, qualifications / experience, relationship with participants, assumptions and / or presuppositions; potential or actual interaction between researchers' characteristics and the research questions, approach, methods, results and / or transferability	8
Context	#7	Setting / site and salient contextual factors; rationale	8
Sampling strategy	#8	How and why research participants, documents, or events were selected; criteria for deciding when no further sampling was necessary (e.g. sampling saturation); rationale	8
Ethical issues pertaining to human subjects	#9	Documentation of approval by an appropriate ethics review board and participant consent, or explanation for lack thereof; other confidentiality and data security issues	5
Data collection methods	#10	Types of data collected; details of data collection procedures including (as appropriate) start and stop dates of data collection and analysis, iterative process, triangulation of sources / methods, and modification of procedures in response to evolving study findings; rationale	8-9
Data collection instruments and technologies	#11	Description of instruments (e.g. interview guides, questionnaires) and devices (e.g. audio recorders) used for data collection; if / how the instruments(s) changed over the course of the study	8-9
Units of study	#12	Number and relevant characteristics of participants, documents, or events included in the study; level of participation (could be	8

For peer review only

		reported in results)	
Data processing	#13	Methods for processing data prior to and during analysis, including transcription, data entry, data management and security, verification of data integrity, data coding, and anonymisation / deidentification of excerpts	8
Data analysis	#14	Process by which inferences, themes, etc. were identified and developed, including the researchers involved in data analysis; usually references a specific paradigm or approach; rationale	9
Techniques to enhance trustworthiness	#15	Techniques to enhance trustworthiness and credibility of data analysis (e.g. member checking, audit trail, triangulation); rationale	9
**Results/findings**			
Syntheses and interpretation	#16	Main findings (e.g. interpretations, inferences, and themes); might include development of a theory or model, or integration with prior research or theory	10-14
Links to empirical data	#17	Evidence (e.g. quotes, field notes, text excerpts, photographs) to substantiate analytic findings	10-14
**Discussion**			
Intergration with prior work, implications, transferability and contribution(s) to the field	#18	Short summary of main findings; explanation of how findings and conclusions connect to, support, elaborate on, or challenge conclusions of earlier scholarship; discussion of scope of application / generalizability; identification of unique contributions(s) to scholarship in a discipline or field	15-17
Limitations	#19	Trustworthiness and limitations of findings	17
**Other**			
Conflicts of interest	#20	Potential sources of influence of perceived influence on study conduct and conclusions; how these were managed	4
Funding	#21	Sources of funding and other support; role of funders in data collection, interpretation and reporting	5

The SRQR checklist is distributed with permission of Wolters Kluwer © 2014 by the Association of American Medical Colleges. This checklist was completed on 15. October 2019 using https://www.goodreports.org/, a tool made by the EQUATOR Network in collaboration with Penelope.ai

For peer review only

BMJ Open

REQUESTS FOR FUTILE TREATMENTS: WHAT MECHANISMS PLAY A ROLE? RESULTS OF A QUALITATIVE STUDY AMONG DUTCH PHYSICIANS

Journal:	*BMJ Open*
Manuscript ID	bmjopen-2019-035675.R1
Article Type:	Original research
Date Submitted by the Author:	29-Jan-2020

Complete List of Authors:	van Bruchem-Visser, Rozemarijn Lidewij; Erasmus Medical Center, internal medicine<br>van Dijk, Gert; Erasmus Medical Center, Medical Ethics and Philosophy of Medicine<br>Mattace Raso, Francesco; Erasmus MC, Section of geriatric medicine, department of Internal Medicine<br>de Beaufort, Inez; Erasmus Medical Center, Medical Ethics and Philosophy of Medicine

**Primary Subject Heading**:	Qualitative research
Secondary Subject Heading:	Ethics, Palliative care, Qualitative research, Health policy
Keywords:	MEDICAL ETHICS, GENERAL MEDICINE (see Internal Medicine), Quality in health care < HEALTH SERVICES ADMINISTRATION & MANAGEMENT, Health policy < HEALTH SERVICES ADMINISTRATION & MANAGEMENT, QUALITATIVE RESEARCH

SCHOLARONE™
Manuscripts

I, the Submitting Author has the right to grant and does grant on behalf of all authors of the Work (as defined in the below author licence), an exclusive licence and/or a non-exclusive licence for contributions from authors who are: i) UK Crown employees; ii) where BMJ has agreed a CC-BY licence shall apply, and/or iii) in accordance with the terms applicable for US Federal Government officers or employees acting as part of their official duties; on a worldwide, perpetual, irrevocable, royalty-free basis to BMJ Publishing Group Ltd ("BMJ") its licensees and where the relevant Journal is co-owned by BMJ to the co-owners of the Journal, to publish the Work in this journal and any other BMJ products and to exploit all rights, as set out in our licence.

The Submitting Author accepts and understands that any supply made under these terms is made by BMJ to the Submitting Author unless you are acting as an employee on behalf of your employer or a postgraduate student of an affiliated institution which is paying any applicable article publishing charge ("APC") for Open Access articles. Where the Submitting Author wishes to make the Work available on an Open Access basis (and intends to pay the relevant APC), the terms of reuse of such Open Access shall be governed by a Creative Commons licence – details of these licences and which Creative Commons licence will apply to this Work are set out in our licence referred to above.

Other than as permitted in any relevant BMJ Author's Self Archiving Policies, I confirm this Work has not been accepted for publication elsewhere, is not being considered for publication elsewhere and does not duplicate material already published. I confirm all authors consent to publication of this Work and authorise the granting of this licence.

For peer review only

Requests for futile treatments: what mechanisms play a role?

Results of a Qualitative Study among Dutch Physicians

Corresponding author:

Rozemarijn Lidewij van Bruchem-Visser MD

Postal address: Department of Internal Medicine, Erasmus MC University Medical Center, PO box 2040 3000 CA Rotterdam, The Netherlands

Email: r.l.visser@erasmusmc.nl

Phone number: +31107035979

Gert van Dijk

Department of Medical Ethics and Philosophy of Medicine, Erasmus MC University Medical Center, Rotterdam, The Netherlands

g.vandijk.2@erasmusmc.nl

Francesco Mattace-Raso MD PhD

Department of Internal Medicine, Erasmus MC University Medical Center, Rotterdam, The Netherlands

f.mattaceraso@erasmusmc.nl

Inez Duconia de Beaufort PhD

Department of Medical Ethics and Philosophy of Medicine, Erasmus MC University Medical Center, Rotterdam, The Netherlands

For peer review only

i.debeaufort@erasmusmc.nl

Keywords: medical ethics, general medicine, quality in health care, health policy, qualitative research

Word count: 3440

Abstract

Objectives:

Overtreatment is increasingly seen as a challenge in clinical practice and can lead to unnecessary interventions, poor healthcare outcomes and increasing costs. However, little is known as to what exactly causes overtreatment. In 2015 the Royal Dutch Medical Association attempted to address this problem and distinguished several mechanisms that were thought to drive overtreatment. In fourteen qualitative interviews among Dutch physicians we investigated which mechanisms played a role in decision-making and whether all mechanisms were considered equally important.

Design:

We asked physicians to present a case from personal experience, in which the patient or family requested continuing treatment against the advice of the physician.

Participants:

Fourteen physicians from five different medical areas agreed to participate.

Setting:

Interviews were held face-to-face at the workplace of the physician.

Results:

From these interviews, it was found that three closely related mechanisms were mentioned most as being a driver of overtreatment, as perceived by the physician: 'death is not a common topic of

For peer review only - http://bmjopen.bmj.com/site/about/guidelines.xhtml

conversation', 'never give up' is the default attitude in our society' and 'patients' culture and outlook on life influences their perception of death'. The mechanism 'medical view taking priority' was mentioned to be an inhibitor of overtreatment.

Conclusions:

Of the fifteen mechanisms described by the report of the Steering Committee of the RDMA, not all mechanisms were mentioned as driving overtreatment. Three mechanisms were mentioned most as being a driver of overtreatment ('death is not a common topic of conversation', 'never give up' is the default attitude in our society' and 'patients' culture and outlook on life influences their perception of death'), some played no role at all, and others were considered to be inhibitors of overtreatment, especially the mechanism 'medical view taking priority'.

For peer review only - http://bmjopen.bmj.com/site/about/guidelines.xhtml

Article summary

Strengths and limitations of this study

- To our knowledge, this is the first time physicians were interviewed on what mechanisms play a role in difficult situations with patients or relatives requesting treatment options the physician deems unnecessary or futile
- A wide variety of patients was described, making the findings applicable to several types of situations and/or physicians
- Relatively small group

Acknowledgements: We would like to thank all participating physicians

Conflict of interests: none of the authors have a conflict of interest.

Author contributions:

RvB has made substantial contributions to the conception or design of the work; she has acquired and interpreted the data for the work. She was involved in drafting the work, approved the final version to be published, and agrees to be accountable for all aspects of the work in ensuring that questions related to the accuracy or integrity of any part of the work are appropriately investigated and resolved.

GvD has made substantial contributions to the conception or design of the work. He was involved in drafting the work, approved the final version to be published, and agrees to be accountable for all aspects of the work in ensuring that questions related to the accuracy or integrity of any part of the work are appropriately investigated and resolved.

FMR has made substantial contributions to the conception or design of the work. He was involved in revising the work critically for important intellectual content, approved the final version to be

For peer review only - http://bmjopen.bmj.com/site/about/guidelines.xhtml

published, and agrees to be accountable for all aspects of the work in ensuring that questions related to the accuracy or integrity of any part of the work are appropriately investigated and resolved.

IdB has made substantial contributions to the conception or design of the work. She was involved in revising the work critically for important intellectual content, approved the final version to be published, and agrees to be accountable for all aspects of the work in ensuring that questions related to the accuracy or integrity of any part of the work are appropriately investigated and resolved.

Data statement: Data are stored on a bit locked computer in the Erasmus Medical Center, Rotterdam

Funding: This research received no specific grant from any funding agency in the public, commercial of not-for-profit sectors.

Ethical approval: Ethical approval was granted by the ethical committee of the Erasmus MC.

For peer review only

Introduction

Overtreatment is increasingly seen as a challenge in clinical practice and can lead to unnecessary interventions, poor healthcare outcomes and increasing costs.(1) The occurrence of overtreatment is acknowledged by both patients and physicians in all patients groups, including the elderly.(2-4)

'Overtreatment' or 'too much medicine' can occur as a result of overdiagnosis, which occurs when people are 'labeled with or treated for a disease that would never cause them harm'. (5, 6)

Overtreatment is defined in different ways: 'treatment that is unnecessary or inappropriate', 'unnecessary investigations and treatment that lack patient benefit or bear the potential to cause harm',(7) treatment that is not 'in line with patient's wishes', 'the provision of medical services for which the potential for harm exceeds the potential for benefit'(8) or 'treatment initiated when there is little or no reliable evidence of a clinically meaningful net benefit, where net benefit equals benefit minus harm'. (9) Overtreatment can concern interventions that can have a positive effect in specific patients , but can harm other patients, for instance percutaneous endoscopic gastrostomy (PEG)-tubes. PEG-tubes can be very beneficiary in some categories of patients, but will harm patients with advanced dementia.(10)

Subjective component

In applying the definition of overtreatment, the patient plays a crucial role. The assessment that a certain intervention is overtreatment in a specific situation can differ between patients, family and physicians. This can make it difficult to objectively determine whether overtreatment has occurred in a specific situation. For instance, what has 'benefit' for a patient can often not objectively be determined as patient preferences differ. Some patients will request as much treatment as possible and accept even a small chance of success and are willing to accept risks, while other patients will be much more reluctant in accepting interventions. Medical interventions can also have benefits other than medical. For instance: an intervention that has little medical chance of success, can still be seen

For peer review only - http://bmjopen.bmj.com/site/about/guidelines.xhtml

For peer review only

as 'useful' by the patient, as it can strengthen the feeling that 'something' or 'everything' has been done.

Foster appropriate care

The Royal Dutch Medical Association (RDMA) established a steering group to address the problem of overtreatment and to 'foster appropriate care for those nearing the end of life'. One of the main tasks of the steering group was to identify mechanisms that are thought to drive overtreatment. In 2015, the Steering Committee for Appropriate End-of-Life Care (SCoAEoLC) published a report 'Just because we can, doesn't mean we should, appropriate end-of-life care.'(11) The report investigated several mechanisms thought to drive overtreatment. These mechanisms play a role in several different domains: society in general, the health care system, industry, professionals, and patients and the public. These mechanisms are in accordance with similar findings from a recent study in Germany.(7) Table 1 shows the fifteen mechanisms that were deemed to be the most important drivers of overtreatment.

Aim of the present study was to research which of the mechanisms as described by the SCoAEoLC were recognized by Dutch physicians of different medical specialties to play a role in driving overtreatment. As the SCoAEoLC has primarily focused on patients in the last phase of life, defined as "being of old age or a suffering from a terminal disease with limited life expectancy", we were wondering whether these mechanisms indeed play a role in the clinical practice of Dutch physicians.

METHODS

Study setting and population

We purposively sampled eighteen physicians differing in years of working experience and medical specialty to participate. Names were randomly selected from the different departments of (academic) hospitals or groups of local general practitioners by using the accessibility guide of the Erasmus MC as well as the list of General Practitioners in the region. Fourteen physicians from five

For peer review only - http://bmjopen.bmj.com/site/about/guidelines.xhtml

different medical areas (internal medicine, general practice (GP), intensive care, surgery, and oncology) agreed to participate. Four physicians were interested in the topic, but were not able to find sufficient time in their schedule to participate. Their work experience ranged from one to thirty-five years. All interviews were conducted between March 2014 and November 2015.

A medical doctor (RvB, internist geriatrician) conducted semi-structured interviews with all physicians , face-to-face at the workplace of the physician. Before the interview, participants were informed that the interview would deal with “disagreement between physician and patient regarding the course of treatment”. Physicians were asked to tell about a case from their own personal experience, in which the patient or family requested continuing or starting of treatment which was not offered or advised by the physician. No limitations were given regarding type of underlying disease, patients’ age, sex or cultural background. After narrating the patient story, the fifteen mechanisms described by the SCoAEoLC (see table 1) were presented and the interviewer asked them whether the mechanism played a role in that case. Participants were given written and verbal information and gave permission to record the interview and store them in a safe location, to be used for verbatim transcription. Ethical approval was granted by the ethical committee of the Erasmus MC.

Data collection and analysis

Interviews were digitally recorded and transcribed verbatim with the interviewees’ permission. Basic demographic information of the described patients (age, sex, ethnicity, religion, relationship with physician) was recorded (see Table 2) as well as of the physicians (sex, medical specialty, years of working experience) (see Table 3). The study was facilitated by QSR NVivo 12 software (QRS International Pty Ltd, Melbourne, Victoria, Australia).

The answers to the mechanisms were coded by two independent researchers (RvB and GvD) to ensure rigorous analysis and to assess whether that mechanism was a factor in the described case.

For peer review only - http://bmjopen.bmj.com/site/about/guidelines.xhtml

Disagreements were settled by consensus. The coding tree was based on the interview guide of the fifteen mechanisms. We used the SRQR checklist when writing our report.(12)

Patient and Public Involvement

Patients or the public were not involved in the design, conduct, or reporting of our research.

For peer review only

For peer review only - http://bmjopen.bmj.com/site/about/guidelines.xhtml

RESULTS

All physicians described a patient facing an end-of-life situation. In five patient stories, the patient was the one requesting a treatment, in the other nine cases the relatives (surrogate decision-makers such as spouses or children) were requesting further treatment. Six patients were aged 25-45 years, three were aged between 45 and 65 years and five were 65 years or older. Physicians explained their choice of patient by different reasons, either the young age of the patient, the acuteness of the situation or the frustration they felt when a treatment was requested or even demanded they had not proposed and felt was unnecessary, futile or even harming the patient.

Fourteen different mechanisms were mentioned to play a role in overtreatment, in total 103 times. This could be as a driver of overtreatment (fourteen different mechanisms, in total 86 times, see figure 1), or as an inhibitor (four different mechanisms, in total 17 times, See figure 2). Mechanism no. 11 discussing possible refusal of treatment is more time-consuming'' was not mentioned as being a driver or inhibitor of overtreatment. The problem of a lack of time was recognized but in all cases physicians explained they took all the time needed.

We found that according to the interviewed physicians, three closely related mechanisms were considered to be the main drivers of overtreatment: no. 1: "death is not a common topic of conversation"; no. 2: "'never give up' is the default attitude in our society" and no. 14: the great unknown: patients' culture and outlook on life influences their perception of death". These three mechanisms were mentioned 37 times as being drivers of overtreatment.

When attributing the perceived drivers of overtreatment to the different parties, there was a distinct difference between the mechanisms. As is shown in figure 1, mechanism no. 1 'death is not a common topic of conversation' was in all cases attributed to the patient or family, whereas mechanism no. 14 'the great unknown: patients' culture and outlook on life influences their

For peer review only - http://bmjopen.bmj.com/site/about/guidelines.xhtml

perception of death' was assigned to the physicians themselves. Mechanism no. 2 "never give up' is the default attitude in our society" was attributed mainly to patients and family, with two cases assigning this mechanism to the physician or a combination of parties.

Of the mechanisms that were not seen as a driver of overtreatment but rather as a potential inhibitor, mechanism nine: 'medical perspectives often still take priority when it comes to making treatment decisions', was most frequently mentioned as an inhibitor (10 times). As is shown in figure 2, this mechanism was in all cases attributed to the physician.

In eleven cases, the physician was not able to communicate directly with the patient, either because the patient spoke a different language and the physician had to communicate by way of the relatives (five cases) or because the patient was incapacitated (six cases).

Death is not a common topic of conversation

The tendency to 'not give up' was considered to be a factor in thirteen out of the fourteen described cases. Interestingly, the difficulties of talking about death and end-of-life topics in general, were in most cases attributed to the patient and family.

"The problem was that, he (the husband of the patient) did not want to talk about his wife dying, because that was just not going to happen." (interview 8)

"The family was not used to talk about the end of life, no. But they found it also hard to talk about bad news in general."(interview 10)

It is sometimes thought physicians themselves find it difficult to talk about death, but this is not what we found in our interviews. Physicians described these kind of conversations as being part of their normal daily routine.

Actually, we talked about it over and over again." (interview 9)

For peer review only - http://bmjopen.bmj.com/site/about/guidelines.xhtml

For peer review only

"Only because she refused to talk about it, not because we were not willing to discuss the subject." (interview 15)

However, two physicians described their own struggles with talking about death. In both cases their hope to cure the patient was the cause of reluctance to talk about a bad outcome.

"So, in this case, while I do possess the skills to talk about it, I found it extremely difficult" (interview 8)

"It caused frustration on my part, and also a feeling of helplessness."(interview 13)

'Never give up' is the default attitude in our society

In our interviews, we found this mechanism was recognized as being an important factor in almost all cases. In thirteen cases physicians described that the patient or family did not want to give up, even when the physician had told them further treatment would be futile.

"This family, most definitely. They wanted us to pull out all the stops, give all possible treatments."(interview 10)

The tendency to not give up, is not something that always arises from the patient, but can sometimes be pushed by relatives, not from a wish to harm the patient, but out of love and empathy:

"Yes. It does play a role. They would rather have her be subjected to a dozen futile treatments than… (...). They were not trying to make memories with their loved one, no, they were still searching the internet for some treatment the doctor had overlooked."(interview 12)

In cases where there was an acute illness, with little time to decide and often little knowledge about the patient due to the acuteness of the situation, physicians described they automatically opted to initially start treatment. To end a treatment, or to not continue on a direction of treatment was described to be difficult.

For peer review only - http://bmjopen.bmj.com/site/about/guidelines.xhtml

"When you get a patient with acute renal failure, we have no time to think about other options. To act is the default position."(interview 6)

"Yes, that did play a role. Especially with the patient, but also with my supervisor, at the start of this process. He was involved in the first part of this case, before I took over. He had been compliant to her wishes so far, had suggested and arranged the percutaneous endoscopic gastrostomy. So I do think this played a role in the first stage of this case." (interview 15)

The great unknown: patients' culture and outlook on life influences their perception of death

Physicians described, sometimes in detail, their unfamiliarity with cultural differences between themselves and (family of) their patients.

"On their part, it was most certainly difficult. Because of religion and culture." (interview 5)

"We found out too late that this daughter had never told her mother the diagnosis, because in their culture, younger family members were not allowed to convey bad news to their elders." (interview 10)

Medical perspectives often still take priority when it comes to making treatment decisions

In our interviews we found physicians used the medical perspective to convince patients not to continue treatment.

"Well yes. I told them I would not perform a CAT scan because there was no medical reason to do so. So I use the medical perspective to explain why I withhold certain interventions."(interview 7)

"In this case the medical perspective led to our decision to tell the patient 'enough is enough'. Locally, we would be able to do a lot of things, but the fact the same problem would come back in different

For peer review only

places, or that the wound would never close, led to us saying 'we will not give any further treatment'. [interview 10]

Taking a medical perspective can therefore mean different things: it can mean the physician takes a perspective in which too much focus is placed on a specific organ, but it can also be an argument not to continue treatment due to medical futility.

For peer review only - http://bmjopen.bmj.com/site/about/guidelines.xhtml

DISCUSSION

In the present study we found that of the fifteen mechanisms described by the SCoAEoLC , not all mechanisms were considered important in driving overtreatment. Three mechanisms were mentioned most as being the drivers of overtreatment ('death is not a common topic of conversation', 'never give up' is the default attitude in our society' and 'patients' culture and outlook on life influences their perception of death'), some played no role at all, and others were considered to be inhibitors of overtreatment, especially the mechanism 'medical view taking priority'.

The three mechanisms that were mentioned most as contributing to overtreatment are closely intertwined. Patients are often hesitant to discuss the approach to end of life and to accept the fact that they are going to die. This reluctance to talk about the end of life is sometimes enhanced by relatives, who can pressure patients not to give up.(13) To talk about death when the patient and family are still aiming for a cure is difficult. Furthermore, in certain cultures it is not customary to discuss the diagnosis, or the approaching death with the patient, making it even harder for the physician to find out what, in a certain case, appropriate care is. As patients and relatives find it difficult to discuss and accept approaching death, physicians often find themselves in a position where patients preferences and values are unknown to them, due to the impossibility to communicate directly with the patient in the acute setting of an end-of-life situation. This was the case in the majority of the narrated patient stories.

The general tendency in society is 'to not give up' in the 'fight' against a certain disease. Dealing with a disease is often seen as a 'fight' or as a 'war'.(14) When dealing with cancer, for instance, patients are supposed to 'fight' the disease. If they are cured they are seen as 'winners' who have 'conquered' the disease. These war-like metaphors are seen also in funding campaigns, such as 'the war on cancer'.

For peer review only - http://bmjopen.bmj.com/site/about/guidelines.xhtml

The cultural and spiritual background of patients can be contributing factors in the perception of physicians that the patient is being subjected to overtreatment. For instance, in some cultures it is not common practice to inform the patients of diagnosis and prognosis. Patients and relatives can also hold specific views on the meaning of suffering and whether pain medication is indicated: 'suffering is purification'. These views can put patients in a situation that physicians find difficult to accept professionally.

As the interviews were held with physicians, the judgment whether the specific treatment was indeed a case of overtreatment was seen from a physician's perspective. It remains unclear whether the patients or relatives also considered the proposed intervention to be a case of overtreatment. This would require further research. The question remains therefore, whether physicians and relatives have similar views on whether a certain intervention is to be considered overtreatment.

In their report, the the SCoAEoLC investigated these mechanisms in various ways, such as conducting literature studies, consultations with experts from a range of disciplines and independent research. However, the report was a consensus document and a wider evidence base is required, especially since the report focused on patients in the last phase of life. It is unknown whether these same mechanisms will apply on other cases of perceived overtreatment.

Interestingly, we found that one of the mechanisms found by Van der Wal et al, the 'medical view taking priority', was not a driver of overtreatment according to the interviewed physicians, but instead was often used as an inhibitor. In our findings the medical 'view, was used by physicians as an argument to refuse to provide the intervention under discussion, as the intervention was deemed medically futile by the physician. The SCoAEoLC considered one of the drivers of overtreatment to be the idea that physicians take too much of a 'medical' perspective, meaning they focus too much at the medical side of a disease, foregoing other important aspects, such as wellbeing of the patients, or social and cultural aspects. According to the SCoAEoLC this means the 'medical perspective often dominates the decision-making process, even when multidisciplinary consultation

For peer review only - http://bmjopen.bmj.com/site/about/guidelines.xhtml

(MDO) is involved. Such bodies often consist only of medical specialists, which can overshadow reflection on social, mental, spiritual, cultural and ideological aspects, as well as general well-being'.(11) However, in our interviews, we found this driver needs to be interpreted in a more nuanced way. In our findings the 'medical view', was used by physicians as an argument to refuse to provide the intervention under discussion, as the intervention was deemed medically futile by the physician.

This might be due to the fact we interviewed physicians, who might have difficulty recognizing this mechanism as playing a role in their relationship with a patient.

Another important finding was that, when asked for a case from their own experience, physicians often presented cases in which the patient was not able to communicate with the physician by himself, either due to a language barrier of mental incapacity. In these cases it was therefore the relatives and not the patient that opposed the advice of the physicians. In the presented cases relatives play a central role in the wish to continue treatment against the advice of the physician. The fact that the wishes of the patient were in all cases unknown to the physician and the relatives enhanced the problem.

One of the reasons for the fact that physicians presented a case in which the relatives played a crucial role might be the subjective aspect of overtreatment. As overtreatment is, at least partly, a subjective phenomenon, physicians might not consider an intervention to be overtreatment when it is the patient him or herself that asks for a specific intervention, as the patient has consented or has specifically asked for the intervention. Patients can have different views on what is appropriate care towards the end of life, and physicians are likely to be willing to go along with the patient – within limits, as the physicians will not likely go along with wishes of the patients that are deemed medically futile - as long as the patient is well informed and is aware of the risks and possible benefits (or lack thereof) of the intervention.

For peer review only - http://bmjopen.bmj.com/site/about/guidelines.xhtml

However, when it is not the patient but relatives that go against the advice of the physician, physicians might sooner consider the intervention to be overtreatment, as they feel the intervention does not serve the medical interest of the patient, the patient has not consented to the procedure, and might actually suffer from it. A refusal of pain medication or sedatives, or a request for a certain intervention will probably be judged in a different manner by the physician if it comes from the patient as opposed to a request from relatives. Although relatives will almost certainly have the best intentions – 'do not let my loved one die' – these intentions might have negative consequences for the patient. It might be acceptable for a physician to see a patient suffer when that patient has deliberately accepted negative consequences of a certain intervention. However, in a mentally incapacitated patient, suffering because of medically futile treatments of any kind is more difficult to accept.

The important role of relatives is not mentioned by the SCoAEoLC as a distinct mechanism that drives overtreatment. This could be explained by the fact that the role of relatives was not a subject of their research. We suggest however, that future research should focus more on the role of relatives in the debate on overtreatment, and the reasons they have for continuation or start of treatments that are deemed inappropriate by the physician. For instance, it would be of interest to find out whether the relatives in these situations considered the intervention to be a case of overtreatment as well, or whether they considered the situation to be appropriate care.

All physicians described a case that had an impact on them, for instance because of the young age of the patient. Interestingly, this is not described as a mechanism of driving overtreatment. However, it was mentioned by several physicians that age did play a role in the perception of the family that all should be done to save the life of the patient.

For peer review only - http://bmjopen.bmj.com/site/about/guidelines.xhtml

Conclusions

We have found that three mechanisms were identified most often as a driving factor in overtreatment: 'death is not a common topic of conversation', 'never give up' is the default attitude in our society' and 'the great unknown: patients' culture and outlook on life influences their perception of death'.

The mechanism 'medical perspectives often still take priority when it comes to making treatment decisions' was mentioned as being an inhibitor of overtreatment in the majority of interviews.

In many cases the relatives play a crucial role in the wish to continue treatment against the advice of the physician. Overtreatment was defined from the perspective of the physicians. Further research is needed to investigate whether relatives of the patient define the same situations as 'overtreatment'.

The findings from this study underline the importance of advance care planning and a timely discussion on patients wishes and preferences regarding the end of life, so as to avoid overtreatment and to foster appropriate care.

For peer review only - http://bmjopen.bmj.com/site/about/guidelines.xhtml

Table 1: Mechanisms described by the SCoAEoLC

1	Death is not a common topic of conversation
2	'Never give up' is the default attitude in our society
3	Action is better than inaction
4	Professional guidelines focus on 'action'
5	Education focuses on 'action'
6	Physicians are payed for treatment
7	With so many care providers and so little coordination, who is responsible?
8	No holistic view of the patient
9	Medical perspectives often still take priority when it comes to making treatment decisions
10	Palliative care is initiated too late
11	Discussing possible refusal of treatment is more time-consuming
12	To talk about death is difficult
13	Uncertainty about what to tell patients
14	The great unknown: patients' culture and outlook on life influences their perception of death
15	People document their wishes and preferences regarding end-of-life care too late, and often not (thorough enough)

For peer review only - http://bmjopen.bmj.com/site/about/guidelines.xhtml

Table 2. Descriptive characteristics of patients

Total number of patients	14
Age	
Aged 25-45	6
Aged 46-65	3
Aged 65 years or older	5
Sex	
Male	4
Female	10
Underlying disease	
Malignancy	5
Kidney disease	2
Diabetes mellitus	0
Neurological condition	4
Chronic Obstructive Pulmonary Disease	0
Infection	0
Complex surgery	1
Medically unexplained	1
Dementia	1
Religious background	
Non-religious/not mentioned	4
Muslim	5
Christian	2
Other	3
Existing relationship patient-physician	7
Treatment requested by patient	5
Treatment requested by one relative	3
Treatment requested by more than one relative	6
Mentally incompetent	6
End-of-life situation	14

For peer review only - http://bmjopen.bmj.com/site/about/guidelines.xhtml

1 Table 3. Descriptive characteristics of the interviewed physicians

Participant	Sex	Specialty	Working experience (years)
1	Female	General practitioner	22
2	Male	Intensive Care	10
3	Female	Oncology	11
4	Male	Surgery	30
5	Female	Intensive Care	35
6	Male	Internal Medicine	4
7	Female	Oncology	20
8	Female	General practitioner	11
9	Female	Internal Medicine	1
10	Female	Internal Medicine	15
11	Male	Surgery	1
12	Male	Surgery	10
13	Female	Oncology	2
14	Female	Intensive Care	6
15	Female	General practitioner	3

2

3

For peer review only - http://bmjopen.bmj.com/site/about/guidelines.xhtml

References

1. Morgan DJ, Wright SM, Dhruva S. Update on medical overuse. JAMA Internal Medicine. 2015;175(1):120-4.
2. How S SA, Lau J, Schoen C. Public Views on US Health System Organization: A Call for New Directions The Commonwealth Fund. 2008(August).
3. Lyu H, Xu T, Brotman D, Mayer-Blackwell B, Cooper M, Daniel M, et al. Overtreatment in the United States. PLoS One. 2017;12(9):e0181970.
4. Thorsteinsdottir B, Swetz KM, Tilburt JC. Dialysis in the frail elderly--a current ethical problem, an impending ethical crisis. J Gen Intern Med. 2013;28(11):1511-6.
5. Morgan DJ, Leppin AL, Smith CD, Korenstein D. A Practical Framework for Understanding and Reducing Medical Overuse: Conceptualizing Overuse Through the Patient-Clinician Interaction. J Hosp Med. 2017;12(5):346-51.
6. Pathirana T, Clark J, Moynihan R. Mapping the drivers of overdiagnosis to potential solutions. BMJ. 2017;358:j3879.
7. Alber K, Kuehlein T, Schedlbauer A, Schaffer S. Medical overuse and quaternary prevention in primary care - A qualitative study with general practitioners. BMC Fam Pract. 2017;18(1):99.
8. Chassin MR, Galvin RW. The urgent need to improve health care quality. Institute of Medicine National Roundtable on Health Care Quality. JAMA. 1998;280(11):1000-5.
9. Herzstein J, Ebell M. Improving quality by doing less: overtreatment. Am Fam Physician. 2015;91(5):289-91.
10. van Bruchem-Visser RL, Mattace-Raso FUS, de Beaufort ID, Kuipers EJ. Percutaneous endoscopic gastrostomy in older patients with and without dementia: Survival and ethical considerations. J Gastroenterol Hepatol. 2019;34(4):736-41.
11. Steering Committee for Appropriate End-of-Life Care. Just because we can, doesn't mean we should. 2015.
12. O'Brien BC, Harris IB, Beckman TJ, Reed DA, Cook DA. Standards for reporting qualitative research: a synthesis of recommendations. Academic Medicine. 2014;89(9):1245-51.
13. Weissman DE. Decision making at a time of crisis near the end of life. JAMA. 2004;292(14):1738-43.
14. Sontag S. Illness as metaphor. London; Allen Lane. 1979.

For peer review only - http://bmjopen.bmj.com/site/about/guidelines.xhtml

Figure 1. Drivers of overtreatment, attributed to different parties

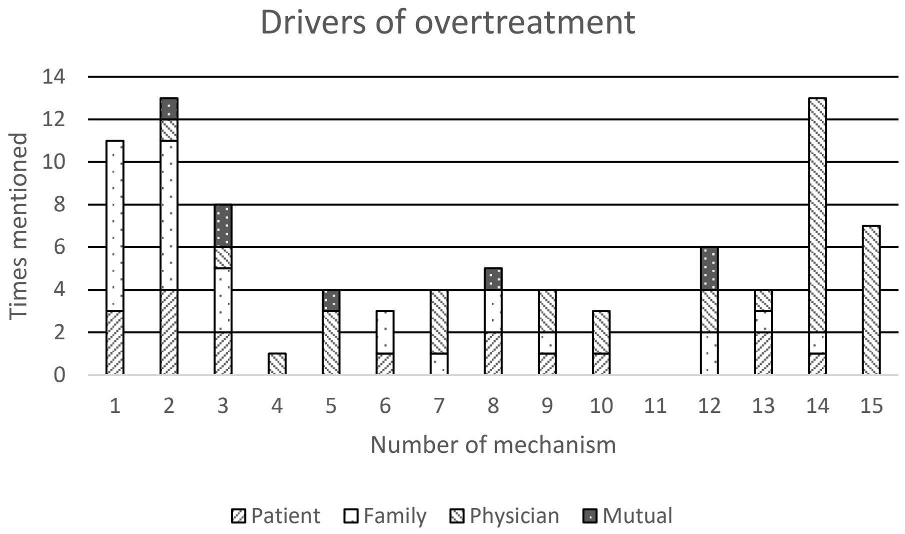

For peer review only

Figure 2. Inhibitors of overtreatment, attributed to different parties

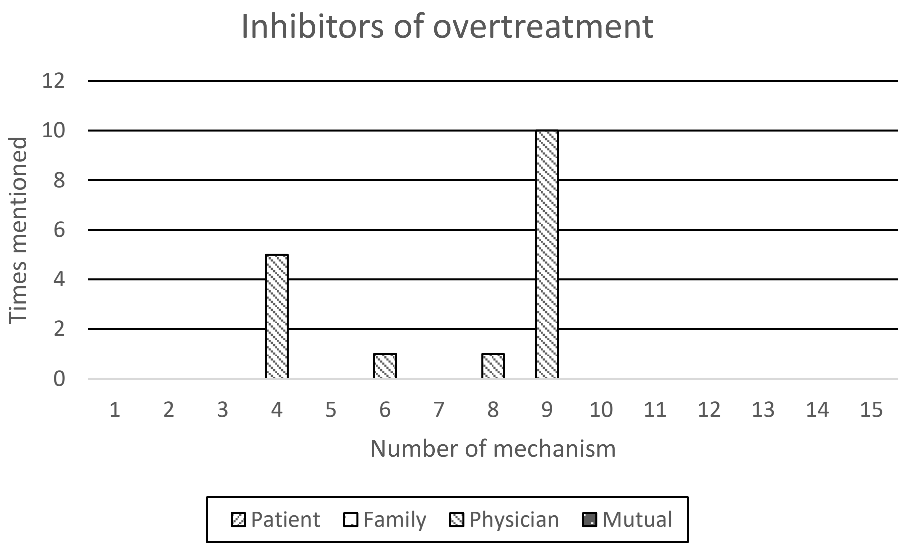

For peer review only

Reporting checklist for qualitative study.

Based on the SRQR guidelines.

Instructions to authors

Complete this checklist by entering the page numbers from your manuscript where readers will find each of the items listed below.

Your article may not currently address all the items on the checklist. Please modify your text to include the missing information. If you are certain that an item does not apply, please write "n/a" and provide a short explanation.

Upload your completed checklist as an extra file when you submit to a journal.

In your methods section, say that you used the SRQRreporting guidelines, and cite them as:

O'Brien BC, Harris IB, Beckman TJ, Reed DA, Cook DA. Standards for reporting qualitative research: a synthesis of recommendations. Acad Med. 2014;89(9):1245-1251.

		Reporting Item	Page Number
**Title**			
	#1	Concise description of the nature and topic of the study identifying the study as qualitative or indicating the approach (e.g. ethnography, grounded theory) or data collection methods (e.g. interview, focus group) is recommended	1
**Abstract**			
	#2	Summary of the key elements of the study using the abstract format of the intended publication; typically includes background, purpose, methods, results and conclusions	2-3
**Introduction**			
Problem formulation	#3	Description and signifcance of the problem / phenomenon studied: review of relevant theory and empirical work; problem statement	6-7
Purpose or research question	#4	Purpose of the study and specific objectives or questions	7

For peer review only

Methods

Qualitative approach and research paradigm	#5	Qualitative approach (e.g. ethnography, grounded theory, case study, phenomenolgy, narrative research) and guiding theory if appropriate; identifying the research paradigm (e.g. postpositivist, constructivist / interpretivist) is also recommended; rationale. The rationale should briefly discuss the justification for choosing that theory, approach, method or technique rather than other options available; the assumptions and limitations implicit in those choices and how those choices influence study conclusions and transferability. As appropriate the rationale for several items might be discussed together.	8
Researcher characteristics and reflexivity	#6	Researchers' characteristics that may influence the research, including personal attributes, qualifications / experience, relationship with participants, assumptions and / or presuppositions; potential or actual interaction between researchers' characteristics and the research questions, approach, methods, results and / or transferability	8
Context	#7	Setting / site and salient contextual factors; rationale	8
Sampling strategy	#8	How and why research participants, documents, or events were selected; criteria for deciding when no further sampling was necessary (e.g. sampling saturation); rationale	8
Ethical issues pertaining to human subjects	#9	Documentation of approval by an appropriate ethics review board and participant consent, or explanation for lack thereof; other confidentiality and data security issues	5
Data collection methods	#10	Types of data collected; details of data collection procedures including (as appropriate) start and stop dates of data collection and analysis, iterative process, triangulation of sources / methods, and modification of procedures in response to evolving study findings; rationale	8-9
Data collection instruments and technologies	#11	Description of instruments (e.g. interview guides, questionnaires) and devices (e.g. audio recorders) used for data collection; if / how the instruments(s) changed over the course of the study	8-9
Units of study	#12	Number and relevant characteristics of participants, documents, or events included in the study; level of participation (could be	8

For peer review only

		reported in results)	
Data processing	#13	Methods for processing data prior to and during analysis, including transcription, data entry, data management and security, verification of data integrity, data coding, and anonymisation / deidentification of excerpts	8
Data analysis	#14	Process by which inferences, themes, etc. were identified and developed, including the researchers involved in data analysis; usually references a specific paradigm or approach; rationale	9
Techniques to enhance trustworthiness	#15	Techniques to enhance trustworthiness and credibility of data analysis (e.g. member checking, audit trail, triangulation); rationale	9
**Results/findings**			
Syntheses and interpretation	#16	Main findings (e.g. interpretations, inferences, and themes); might include development of a theory or model, or integration with prior research or theory	10-14
Links to empirical data	#17	Evidence (e.g. quotes, field notes, text excerpts, photographs) to substantiate analytic findings	10-14
**Discussion**			
Intergration with prior work, implications, transferability and contribution(s) to the field	#18	Short summary of main findings; explanation of how findings and conclusions connect to, support, elaborate on, or challenge conclusions of earlier scholarship; discussion of scope of application / generalizability; identification of unique contributions(s) to scholarship in a discipline or field	15-17
Limitations	#19	Trustworthiness and limitations of findings	17
**Other**			
Conflicts of interest	#20	Potential sources of influence of perceived influence on study conduct and conclusions; how these were managed	4
Funding	#21	Sources of funding and other support; role of funders in data collection, interpretation and reporting	5

The SRQR checklist is distributed with permission of Wolters Kluwer © 2014 by the Association of American Medical Colleges. This checklist was completed on 15. October 2019 using https://www.goodreports.org/, a tool made by the EQUATOR Network in collaboration with Penelope.ai

For peer review only

BMJ Open

REQUESTS FOR FUTILE TREATMENTS: WHAT MECHANISMS PLAY A ROLE? RESULTS OF A QUALITATIVE STUDY AMONG DUTCH PHYSICIANS

Journal:	*BMJ Open*
Manuscript ID	bmjopen-2019-035675.R2
Article Type:	Original research
Date Submitted by the Author:	23-Mar-2020
Complete List of Authors:	van Bruchem-Visser, Rozemarijn Lidewij; Erasmus Medical Center, internal medicine<br>van Dijk, Gert; Erasmus Medical Center, Medical Ethics and Philosophy of Medicine<br>Mattace Raso, Francesco; Erasmus MC, Section of geriatric medicine, department of Internal Medicine<br>de Beaufort, Inez; Erasmus Medical Center, Medical Ethics and Philosophy of Medicine
**Primary Subject Heading**:	Qualitative research
Secondary Subject Heading:	Ethics, Palliative care, Qualitative research, Health policy
Keywords:	MEDICAL ETHICS, GENERAL MEDICINE (see Internal Medicine), Quality in health care < HEALTH SERVICES ADMINISTRATION & MANAGEMENT, Health policy < HEALTH SERVICES ADMINISTRATION & MANAGEMENT, QUALITATIVE RESEARCH

SCHOLARONE™
Manuscripts

I, the Submitting Author has the right to grant and does grant on behalf of all authors of the Work (as defined in the below author licence), an exclusive licence and/or a non-exclusive licence for contributions from authors who are: i) UK Crown employees; ii) where BMJ has agreed a CC-BY licence shall apply, and/or iii) in accordance with the terms applicable for US Federal Government officers or employees acting as part of their official duties; on a worldwide, perpetual, irrevocable, royalty-free basis to BMJ Publishing Group Ltd ("BMJ") its licensees and where the relevant Journal is co-owned by BMJ to the co-owners of the Journal, to publish the Work in this journal and any other BMJ products and to exploit all rights, as set out in our licence.

The Submitting Author accepts and understands that any supply made under these terms is made by BMJ to the Submitting Author unless you are acting as an employee on behalf of your employer or a postgraduate student of an affiliated institution which is paying any applicable article publishing charge ("APC") for Open Access articles. Where the Submitting Author wishes to make the Work available on an Open Access basis (and intends to pay the relevant APC), the terms of reuse of such Open Access shall be governed by a Creative Commons licence – details of these licences and which Creative Commons licence will apply to this Work are set out in our licence referred to above.

Other than as permitted in any relevant BMJ Author's Self Archiving Policies, I confirm this Work has not been accepted for publication elsewhere, is not being considered for publication elsewhere and does not duplicate material already published. I confirm all authors consent to publication of this Work and authorise the granting of this licence.

For peer review only

Requests for futile treatments: what mechanisms play a role?

Results of a Qualitative Study among Dutch Physicians

Corresponding author:

Rozemarijn Lidewij van Bruchem-Visser MD

Postal address: Department of Internal Medicine, Erasmus MC University Medical Center, PO box 2040 3000 CA Rotterdam, The Netherlands

Email: r.l.visser@erasmusmc.nl

Phone number: +31107035979

Gert van Dijk

Department of Medical Ethics and Philosophy of Medicine, Erasmus MC University Medical Center, Rotterdam, The Netherlands

g.vandijk.2@erasmusmc.nl

Francesco Mattace-Raso MD PhD

Department of Internal Medicine, Erasmus MC University Medical Center, Rotterdam, The Netherlands

f.mattaceraso@erasmusmc.nl

Inez Duconia de Beaufort PhD

Department of Medical Ethics and Philosophy of Medicine, Erasmus MC University Medical Center, Rotterdam, The Netherlands

For peer review only - http://bmjopen.bmj.com/site/about/guidelines.xhtml

i.debeaufort@erasmusmc.nl

Keywords: medical ethics, general medicine, quality in health care, health policy, qualitative research

Word count: 3440

Abstract

Objectives:

Overtreatment is increasingly seen as a challenge in clinical practice and can lead to unnecessary interventions, poor healthcare outcomes and increasing costs. However, little is known as to what exactly causes overtreatment. In 2015 the Royal Dutch Medical Association attempted to address this problem and distinguished several mechanisms that were thought to drive overtreatment. In fourteen qualitative interviews among Dutch physicians we investigated which mechanisms played a role in decision-making and whether all mechanisms were considered equally important.

Design:

We asked physicians to present a case from personal experience, in which the patient or family requested continuing treatment against the advice of the physician.

Participants:

Fourteen physicians from five different medical areas agreed to participate.

Setting:

Interviews were held face-to-face at the workplace of the physician.

Results:

From these interviews, it was found that three closely related mechanisms were mentioned most as being a driver of overtreatment, as perceived by the physician: 'death is not a common topic of

For peer review only

conversation', 'never give up' is the default attitude in our society' and 'patients' culture and outlook on life influences their perception of death'. The mechanism 'medical view taking priority' was mentioned to be an inhibitor of overtreatment.

Conclusions:

Of the fifteen mechanisms described by the report of the Steering Committee of the RDMA, not all mechanisms were mentioned as driving overtreatment. Three mechanisms were mentioned most as being a driver of overtreatment ('death is not a common topic of conversation'; 'never give up' is the default attitude in our society' and 'patients' culture and outlook on life influences their perception of death'), some played no role at all, and others were considered to be inhibitors of overtreatment, especially the mechanism 'medical view taking priority'.

For peer review only

ARTICLE SUMMARY

STRENGTHS AND LIMITATIONS OF THIS STUDY

- Physicians from different specialties and working experiences were interviewed, making the findings more generalizable.
- A wide variety of patients facing end-of-life decisions was described, making the findings applicable to several types of situations and/or physicians
- All interviewed physicians work in The Netherlands. Opinions on what futile treatment is, might differ between different countries of cultures.

ACKNOWLEDGEMENTS: We would like to thank all participating physicians

CONFLICT OF INTERESTS: none of the authors have a conflict of interest.

AUTHOR CONTRIBUTIONS:

RvB has made substantial contributions to the conception or design of the work She has acquired and interpreted the data for the work. She was involved in drafting the work, approved the final version to be published, and agrees to be accountable for all aspects of the work in ensuring that questions related to the accuracy or integrity of any part of the work are appropriately investigated and resolved.

GvD has made substantial contributions to the conception or design of the work. He was involved in drafting the work, approved the final version to be published, and agrees to be accountable for all aspects of the work in ensuring that questions related to the accuracy or integrity of any part of the work are appropriately investigated and resolved.

FMR has made substantial contributions to the conception or design of the work. He was involved in revising the work critically for important intellectual content, approved the final version to be

For peer review only

published, and agrees to be accountable for all aspects of the work in ensuring that questions related to the accuracy or integrity of any part of the work are appropriately investigated and resolved.

IdB has made substantial contributions to the conception or design of the work. She was involved in revising the work critically for important intellectual content, approved the final version to be published, and agrees to be accountable for all aspects of the work in ensuring that questions related to the accuracy or integrity of any part of the work are appropriately investigated and resolved.

Data statement: Data are stored on a bit locked computer in the Erasmus Medical Center, Rotterdam

Funding: This research received no specific grant from any funding agency in the public, commercial of not-for-profit sectors.

Ethical approval: The ethical committee of the Erasmus MC was consulted who confirmed that this study is not subject to the Medical Research Involving Human Subjects act.

For peer review only - http://bmjopen.bmj.com/site/about/guidelines.xhtml

INTRODUCTION

Overtreatment is increasingly seen as a challenge in clinical practice and can lead to unnecessary interventions, poor healthcare outcomes and increasing costs.(1) The occurrence of overtreatment is acknowledged by both patients and physicians in all patients groups, including the elderly.(2-4)

'Overtreatment' or 'too much medicine' can occur as a result of overdiagnosis, which occurs when people are 'labeled with or treated for a disease that would never cause them harm'. (5, 6)

Overtreatment is defined in different ways: 'treatment that is unnecessary or inappropriate'; 'unnecessary investigations and treatment that lack patient benefit or bear the potential to cause harm';(7) treatment that is not 'in line with patient's wishes'; 'the provision of medical services for which the potential for harm exceeds the potential for benefit'(8) or 'treatment initiated when there is little or no reliable evidence of a clinically meaningful net benefit, where net benefit equals benefit minus harm'. (9) Overtreatment can concern interventions that can have a positive effect in specific patients , but can harm other patients, for instance percutaneous endoscopic gastrostomy (PEG)-tubes. PEG-tubes can be very beneficiary in some categories of patients, but will harm patients with advanced dementia.(10)

For peer review only

Subjective component

In applying the definition of overtreatment, the patient plays a crucial role. The assessment that a certain intervention is overtreatment in a specific situation can differ between patients, family and physicians. This can make it difficult to objectively determine whether overtreatment has occurred in a specific situation. For instance, what has 'benefit' for a patient can often not objectively be determined as patient preferences differ. Some patients will request as much treatment as possible and accept even a small chance of success and are willing to accept risks, while other patients will be much more reluctant in accepting interventions. Medical interventions can also have other benefits .

For peer review only - http://bmjopen.bmj.com/site/about/guidelines.xhtml

For instance: an intervention that has little medical chance of success, can still be seen as 'useful' by the patient, as it can strengthen the feeling that 'something' or 'everything' has been done.

Foster appropriate care

The Royal Dutch Medical Association (RDMA) established a steering group to address the problem of overtreatment and to 'foster appropriate care for those nearing the end of life'. One of the main tasks of the steering group was to identify mechanisms that are thought to drive overtreatment. In 2015, the Steering Committee for Appropriate End-of-Life Care (SCoAEoLC) published a report 'Just because we can, doesn't mean we should, appropriate end-of-life care.'(11) The report investigated several mechanisms thought to drive overtreatment. These mechanisms play a role in several different domains: society in general, the health care system, industry, professionals, and patients and the public. These mechanisms are in accordance with similar findings from a recent study in Germany.(7) Table 1 shows the fifteen mechanisms that were deemed to be the most important drivers of overtreatment.

Aim of the present study was to research which of the mechanisms as described by the SCoAEoLC were recognized by Dutch physicians of different medical specialties to play a role in driving overtreatment. As the SCoAEoLC has primarily focused on patients in the last phase of life, defined as "being of old age or a suffering from a terminal disease with limited life expectancy", we were wondering whether these mechanisms do play a role in the clinical practice of Dutch physicians.

Methods

Study setting and population

We purposively sampled eighteen physicians differing in years of working experience and medical specialty to participate. Names were randomly selected from the different departments of (academic) hospitals or groups of local general practitioners by using the accessibility guide of the Erasmus MC as well as the list of General Practitioners in the region. Fourteen physicians from five

For peer review only - http://bmjopen.bmj.com/site/about/guidelines.xhtml

different medical areas (internal medicine, general practice (GP), intensive care, surgery, and oncology) agreed to participate. Four physicians were interested in the topic, but were not able to find sufficient time in their schedule to participate. Their work experience ranged from one to thirty-five years. All interviews were conducted between March 2014 and November 2015.

A medical doctor (RvB, internist geriatrician) conducted semi-structured interviews with all physicians , face-to-face at the workplace of the physician. Before the interview, participants were informed that the interview would deal with “disagreement between physician and patient regarding the course of treatment”. Physicians were asked to tell about a case from their own personal experience, in which the patient or family requested continuing or starting of treatment which was not offered or advised by the physician. No limitations were given regarding type of underlying disease, patients’ age, sex or cultural background. After narrating the patient’s story, the fifteen mechanisms described by the SCoAEoLC (see table 1) were presented and the interviewer asked them whether the mechanism played a role in that case. Participants were given written and verbal information and gave permission to record the interview and store them in a safe location, to be used for verbatim transcription. Ethical approval was granted by the ethical committee of the Erasmus MC.

Data collection and analysis

Interviews were digitally recorded and transcribed verbatim with the interviewees’ permission. Basic demographic information of the described patients (age, sex, ethnicity, religion, relationship with physician) was recorded (see Table 2) as well as of the physicians (sex, medical specialty, years of working experience) (see Table 3). The study was facilitated by QSR NVivo 12 software (QRS International Pty Ltd, Melbourne, Victoria, Australia).

The answers to the mechanisms were coded by two independent researchers (RvB and GvD) to ensure rigorous analysis and to assess whether that mechanism was a factor in the described case.

For peer review only - http://bmjopen.bmj.com/site/about/guidelines.xhtml

Disagreements were settled by consensus. The coding tree was based on the interview guide of the fifteen mechanisms. We used the SRQR checklist when writing our report.(12)

Patient and Public Involvement

Patients or the public were not involved in the design, conduct, or reporting of our research.

For peer review only

For peer review only - http://bmjopen.bmj.com/site/about/guidelines.xhtml

RESULTS

All physicians described a patient facing an end-of-life situation. In five patients' stories, the patient was the one requesting a treatment, in the other nine cases the relatives (surrogate decision-makers such as spouses or children) were requesting further treatment. Six patients were aged between 25 and 45 years, three between 45 and 65 and five were 65 years or older. Physicians explained their choice of patient by different reasons, either the young age of the patient, the acuteness of the situation or the frustration they felt when a treatment was requested or even demanded they had not proposed and felt was unnecessary, futile or even harming the patient.

Fourteen different mechanisms were mentioned to play a role in overtreatment, in total 103 times. This could be as a driver of overtreatment (fourteen different mechanisms, in total 86 times, see figure 1), or as an inhibitor (four different mechanisms, in total 17 times, see figure 2). Mechanism no. 11 discussing possible refusal of treatment is more time-consuming'' was not mentioned as being a driver or inhibitor of overtreatment. The problem of a lack of time was recognized but in all cases physicians explained they took all the time needed.

We found that according to the interviewed physicians, three closely related mechanisms were considered to be the main drivers of overtreatment: no. 1: "death is not a common topic of conversation"; no. 2: "never give up' is the default attitude in our society" and no. 14: the great unknown: patients' culture and outlook on life influences their perception of death". These three mechanisms were mentioned 37 times as being drivers of overtreatment.

When attributing the perceived drivers of overtreatment to the different parties, there was a distinct difference between the mechanisms. As is shown in figure 1, mechanism no. 1 'death is not a common topic of conversation' was in all cases attributed to the patient or family, whereas mechanism no. 14 'the great unknown: patients' culture and outlook on life influences their

For peer review only - http://bmjopen.bmj.com/site/about/guidelines.xhtml

perception of death' was assigned to the physicians themselves. Mechanism no. 2 "never give up' is the default attitude in our society" was attributed mainly to patients and family, with two cases assigning this mechanism to the physician or a combination of parties.

Of the mechanisms that were not seen as a driver of overtreatment but rather as a potential inhibitor, mechanism nine: 'medical perspectives often still take priority when it comes to making treatment decisions', was most frequently mentioned as an inhibitor (10 times). As is shown in figure 2, this mechanism was in all cases attributed to the physician.

In eleven cases, the physician was not able to communicate directly with the patient, either because the patient spoke a different language and the physician had to communicate by way of the relatives (five cases) or because the patient was incapacitated (six cases).

Death is not a common topic of conversation

The tendency to 'not give up' was considered to be a factor in thirteen out of the fourteen described cases. The difficulties of talking about death and end-of-life topics in general, were in most cases attributed to the patient and family.

"The problem was that, he (the husband of the patient) did not want to talk about his wife dying, because that was just not going to happen." (interview 8)

"The family was not used to talk about the end of life, no. But they found it also hard to talk about bad news in general."(interview 10)

It is sometimes thought physicians themselves find it difficult to talk about death, but this is not what we found in our interviews. Physicians described these kind of conversations as being part of their normal daily routine.

"Actually, we talked about it over and over again." (interview 9)

For peer review only - http://bmjopen.bmj.com/site/about/guidelines.xhtml

For peer review only

"Only because she refused to talk about it, not because we were not willing to discuss the subject." (interview 15)

However, two physicians described their own struggles with talking about death. In both cases their hope to cure the patient was the cause of reluctance to talk about a bad outcome.

"So, in this case, while I do possess the skills to talk about it, I found it extremely difficult" (interview 8)

"It caused frustration on my part, and also a feeling of helplessness."(interview 13)

'Never give up' is the default attitude in our society

In our interviews, we found this mechanism was recognized as being an important factor in almost all cases. In thirteen cases physicians described that the patient or family did not want to give up, even when the physician had told them further treatment would be futile.

"This family, most definitely. They wanted us to pull out all the stops, give all possible treatments."(interview 10)

The tendency to not give up, is not something that always arises from the patient, but can sometimes be pushed by relatives, not from a wish to harm the patient, but out of love and empathy:

"Yes. It does play a role. They would rather have her be subjected to a dozen futile treatments than... (...). They were not trying to make memories with their loved one, no, they were still searching the internet for some treatment the doctor had overlooked."(interview 12)

In cases where there was an acute illness, with little time to decide and often little knowledge about the patient due to the acuteness of the situation, physicians described they automatically opted to initially start treatment. To end a treatment, or to not continue on a direction of treatment was described to be difficult.

For peer review only - http://bmjopen.bmj.com/site/about/guidelines.xhtml

"When you get a patient with acute renal failure, we have no time to think about other options. To act is the default position."(interview 6)

"Yes, that did play a role. Especially with the patient, but also with my supervisor, at the start of this process. He was involved in the first part of this case, before I took over. He had been compliant to her wishes so far, had suggested and arranged the percutaneous endoscopic gastrostomy. So I do think this played a role in the first stage of this case." (interview 15)

The great unknown: patients' culture and outlook on life influences their perception of death

Physicians described, sometimes in detail, their unfamiliarity with cultural differences between themselves and (family/relatives of) their patients.

"On their part, it was most certainly difficult. Because of religion and culture." (interview 5)

"We found out too late that this daughter had never told her mother the diagnosis, because in their culture, younger family members were not allowed to convey bad news to their elders." (interview 10)

Medical perspectives often still take priority when it comes to making treatment decisions

In our interviews we found physicians used the medical perspective to convince patients not to continue treatment.

"Well yes. I told them I would not perform a CAT scan because there was no medical reason to do so. So I use the medical perspective to explain why I withhold certain interventions."(interview 7)

"In this case the medical perspective led to our decision to tell the patient 'enough is enough'. Locally, we would be able to do a lot of things, but the fact the same problem would come back in different

For peer review only - http://bmjopen.bmj.com/site/about/guidelines.xhtml

places, or that the wound would never close, led to us saying ‘we will not give any further treatment’. [interview 10]

Taking a medical perspective can therefore mean different things: it can mean the physician takes a perspective in which too much focus is placed on a specific organ, but it can also be an argument not to continue treatment due to medical futility.

For peer review only

For peer review only - http://bmjopen.bmj.com/site/about/guidelines.xhtml

Discussion

In the present study we found that of the fifteen mechanisms described by the SCoAEoLC , not all mechanisms were considered important in driving overtreatment. Three mechanisms were mentioned most as being the drivers of overtreatment (‘death is not a common topic of conversation’, ‘never give up’ is the default attitude in our society’ and ‘patients' culture and outlook on life influences their perception of death’), some played no role at all, and others were considered to be inhibitors of overtreatment, especially the mechanism ‘medical view taking priority’.

The three mechanisms that were mentioned most as contributing to overtreatment are closely intertwined.

Patients are often hesitant to discuss the approach to end of life and to accept the fact that they are going to die. This reluctance to talk about the end of life is sometimes enhanced by relatives, who can pressure patients not to give up.(13) To talk about death when the patient and family are still aiming for a cure is difficult. Furthermore, in certain cultures it is not customary to discuss the diagnosis, or the approaching death with the patient, making it even harder for the physician to find out what, in a certain case, appropriate care is. As patients and relatives find it difficult to discuss and accept approaching death, physicians often find themselves in a position where patients preferences and values are unknown to them, due to the impossibility to communicate directly with the patient in the acute setting of an end-of-life situation. This was the case in the majority of the narrated patient stories.

The general tendency in society is ‘to not give up’ in the ‘fight’ against a certain disease. Dealing with a disease is often seen as a ‘fight’ or as a ‘war’.(14) When dealing with cancer, for instance, patients are supposed to ‘fight’ the disease. If they are cured they are seen as ‘winners’ who have ‘conquered’ the disease. These war-like metaphors are also seen in funding campaigns, such as ‘the war on cancer’.

For peer review only - http://bmjopen.bmj.com/site/about/guidelines.xhtml

The cultural and spiritual background of patients can be contributing factors in the perception of physicians that the patient is being subjected to overtreatment. For instance, in some cultures it is not common practice to inform the patients of diagnosis and prognosis. Patients and relatives can also hold specific views on the meaning of suffering and whether pain medication is indicated: 'suffering is purification'. These views can put patients in a situation that physicians find difficult to accept professionally.

As the interviews were held with physicians, the judgment whether the specific treatment was indeed a case of overtreatment was seen from a physician's perspective. It remains unclear whether the patients or relatives also considered the proposed intervention to be a case of overtreatment. This would require further research. The question remains therefore, whether physicians and relatives have similar views on whether a certain intervention is to be considered overtreatment.

In their report, the the SCoAEoLC investigated these mechanisms in various ways, such as conducting literature studies, consultations with experts from a range of disciplines and independent research. However, the report was a consensus document and a wider evidence base is required, especially since the report focused on patients in the last phase of life. It is unknown whether these same mechanisms will apply to other cases of perceived overtreatment.

Interestingly, we found that one of the mechanisms found by Van der Wal et al; the 'medical view taking priority', was not a driver of overtreatment according to the interviewed physicians, but was often used as an inhibitor instead. In our findings the medical 'view, was used by physicians as an argument to refuse to provide the intervention under discussion, as the intervention was deemed medically futile by the physicianThe SCoAEoLC considered one of the drivers of overtreatment to be the idea that physicians take too much of a 'medical' perspective, meaning they focus too much on the medical side of a disease, foregoing other important aspects, such as wellbeing of the patients, or social and cultural aspects. According to the SCoAEoLC this means the 'medical perspective often dominates the decision-making process, even when multidisciplinary consultation (MDO) is involved.

For peer review only - http://bmjopen.bmj.com/site/about/guidelines.xhtml

Such bodies often consist only of medical specialists, which can overshadow reflection on social, mental, spiritual, cultural and ideological aspects, as well as general well-being'.(11) However, in our interviews, we found this driver needs to be interpreted in a more nuanced way. In our findings the 'medical view', was used by physicians as an argument to refuse to provide the intervention under discussion, as the intervention was deemed medically futile by the physician.

This might be due to the fact we interviewed physicians, who might have difficulty recognizing this mechanism as playing a role in their relationship with a patient.

Another important finding was that, when asked for a case from their own experience, physicians often presented cases in which the patient was not able to communicate with the physician by her/himself, either due to a language barrier of mental incapacity. In these cases it was therefore the relatives and not the patient that opposed the advice of the physicians. In the presented cases relatives play a central role in the wish to continue treatment against the advice of the physician. The fact that the wishes of the patient were in all cases unknown to the physician and the relatives enhanced the problem.

One of the reasons for the fact that physicians presented a case in which the relatives played a crucial role might be the subjective aspect of overtreatment. As overtreatment is, at least partly, a subjective phenomenon, physicians might not consider an intervention to be overtreatment when it is the patient him- or herself that asks for a specific intervention, as the patient has consented or has specifically asked for the intervention. Patients can have different views on what is appropriate care towards the end of life, and physicians are likely to be willing to go along with the patient – within limits, as the physicians will not likely go along with wishes of the patients that are deemed medically futile - as long as the patient is well informed and is aware of the risks and possible benefits (or lack thereof) of the intervention.

For peer review only - http://bmjopen.bmj.com/site/about/guidelines.xhtml

However, when it is not the patient but relatives that go against the advice of the physician, physicians might sooner consider the intervention to be overtreatment, as they feel the intervention does not serve the medical interest of the patient, the patient has not consented to the procedure, and might actually suffer from it. A refusal of pain medication or sedatives, or a request for a certain intervention will probably be judged in a different manner by the physician if it comes from the patient as opposed to a request from relatives. Although relatives will almost certainly have the best intentions – 'do not let my loved one die' – these intentions might have negative consequences for the patient. It might be acceptable for a physician to see a patient suffer when that patient has deliberately accepted negative consequences of a certain intervention. However, in a mentally incapacitated patient, suffering because of medically futile treatments of any kind is more difficult to accept.

The important role of relatives is not mentioned by the SCoAEoLC as a distinct mechanism that drives overtreatment. This could be explained by the fact that the role of relatives was not a subject of their research. We suggest however, that future research should focus more on the role of relatives in the debate on overtreatment, and the reasons they have for continuation or start of treatments that are deemed inappropriate by the physician. For instance, it would be of interest to find out whether the relatives in these situations considered the intervention to be a case of overtreatment as well, or whether they considered the situation to be appropriate care.

All physicians described a case that had an impact on them. The described patients were generally younger individuals and the decisions to be made were related to end-of-life decision making. Interestingly, (younger) age is not described as a mechanism of driving overtreatment. However, it was mentioned by several physicians that age did play a role in the perception of the family/relatives that all should be done to save the patient's life.

For peer review only

CONCLUSIONS

We have found that three mechanisms were identified most often as a driving factor in overtreatment: 'death is not a common topic of conversation', 'never give up' is the default attitude in our society' and 'the great unknown: patients' culture and outlook on life influences their perception of death'.

The mechanism "medical perspectives often still take priority when it comes to making treatment decisions" was mentioned as being an inhibitor of overtreatment in the majority of interviews.

In many cases the relatives play a crucial role in the wish to continue treatment against the advice of the physician. Overtreatment was defined from the perspective of the physicians. Further research is needed to investigate whether relatives of the patient define the same situations as 'overtreatment'.

The findings from this study underline the importance of advance care planning and a timely discussion on patients' wishes and preferences regarding the end of life, so as to avoid overtreatment and to foster appropriate care.

For peer review only

Table 1: Mechanisms described by the SCoAEoLC

1	Death is not a common topic of conversation
2	'Never give up' is the default attitude in our society
3	Action is better than inaction
4	Professional guidelines focus on 'action'
5	Education focuses on 'action'
6	Physicians are payed for treatment
7	With so many care providers and so little coordination, who is responsible?
8	No holistic view of the patient
9	Medical perspectives often still take priority when it comes to making treatment decisions
10	Palliative care is initiated too late
11	Discussing possible refusal of treatment is more time-consuming
12	To talk about death is difficult
13	Uncertainty about what to tell patients
14	The great unknown: patients' culture and outlook on life influences their perception of death
15	People document their wishes and preferences regarding end-of-life care too late, and often not (thorough enough)

For peer review only - http://bmjopen.bmj.com/site/about/guidelines.xhtml

Table 2. Descriptive characteristics of patients

Total number of patients	14
Age	
Aged 25-45	6
Aged 46-65	3
Aged 65 years or older	5
Sex	
Male	4
Female	10
Underlying disease	
Malignancy	5
Kidney disease	2
Diabetes mellitus	0
Neurological condition	4
Chronic Obstructive Pulmonary Disease	0
Infection	0
Complex surgery	1
Medically unexplained	1
Dementia	1
Religious background	
Non-religious/not mentioned	4
Muslim	5
Christian	2
Other	3
Existing relationship patient-physician	7
Treatment requested by patient	5
Treatment requested by one relative	3
Treatment requested by more than one relative	6
Mentally incompetent	6
End-of-life situation	14

For peer review only - http://bmjopen.bmj.com/site/about/guidelines.xhtml

Table 3. Descriptive characteristics of the interviewed physicians

Participant	Sex	Specialty	Working experience (years)
1	Female	General practitioner	22
2	Male	Intensive Care	10
3	Female	Oncology	11
4	Male	Surgery	30
5	Female	Intensive Care	35
6	Male	Internal Medicine	4
7	Female	Oncology	20
8	Female	General practitioner	11
9	Female	Internal Medicine	1
10	Female	Internal Medicine	15
11	Male	Surgery	1
12	Male	Surgery	10
13	Female	Oncology	2
14	Female	Intensive Care	6
15	Female	General practitioner	3

For peer review only

For peer review only - http://bmjopen.bmj.com/site/about/guidelines.xhtml

References

1. Morgan DJ, Wright SM, Dhruva S. Update on medical overuse. JAMA Internal Medicine. 2015;175(1):120-4.
2. How S SA, Lau J, Schoen C. Public Views on US Health System Organization: A Call for New Directions The Commonwealth Fund. 2008(August).
3. Lyu H, Xu T, Brotman D, Mayer-Blackwell B, Cooper M, Daniel M, et al. Overtreatment in the United States. PLoS One. 2017;12(9):e0181970.
4. Thorsteinsdottir B, Swetz KM, Tilburt JC. Dialysis in the frail elderly--a current ethical problem, an impending ethical crisis. J Gen Intern Med. 2013;28(11):1511-6.
5. Morgan DJ, Leppin AL, Smith CD, Korenstein D. A Practical Framework for Understanding and Reducing Medical Overuse: Conceptualizing Overuse Through the Patient-Clinician Interaction. J Hosp Med. 2017;12(5):346-51.
6. Pathirana T, Clark J, Moynihan R. Mapping the drivers of overdiagnosis to potential solutions. BMJ. 2017;358:j3879.
7. Alber K, Kuehlein T, Schedlbauer A, Schaffer S. Medical overuse and quaternary prevention in primary care - A qualitative study with general practitioners. BMC Fam Pract. 2017;18(1):99.
8. Chassin MR, Galvin RW. The urgent need to improve health care quality. Institute of Medicine National Roundtable on Health Care Quality. JAMA. 1998;280(11):1000-5.
9. Herzstein J, Ebell M. Improving quality by doing less: overtreatment. Am Fam Physician. 2015;91(5):289-91.
10. van Bruchem-Visser RL, Mattace-Raso FUS, de Beaufort ID, Kuipers EJ. Percutaneous endoscopic gastrostomy in older patients with and without dementia: Survival and ethical considerations. J Gastroenterol Hepatol. 2019;34(4):736-41.
11. Steering Committee for Appropriate End-of-Life Care. Just because we can, doesn't mean we should. 2015.
12. O'Brien BC, Harris IB, Beckman TJ, Reed DA, Cook DA. Standards for reporting qualitative research: a synthesis of recommendations. Academic Medicine. 2014;89(9):1245-51.
13. Weissman DE. Decision making at a time of crisis near the end of life. JAMA. 2004;292(14):1738-43.
14. Sontag S. Illness as metaphor. London; Allen Lane. 1979.

For peer review only

For peer review only - http://bmjopen.bmj.com/site/about/guidelines.xhtml

Figure 1. Drivers of overtreatment, attributed to different parties

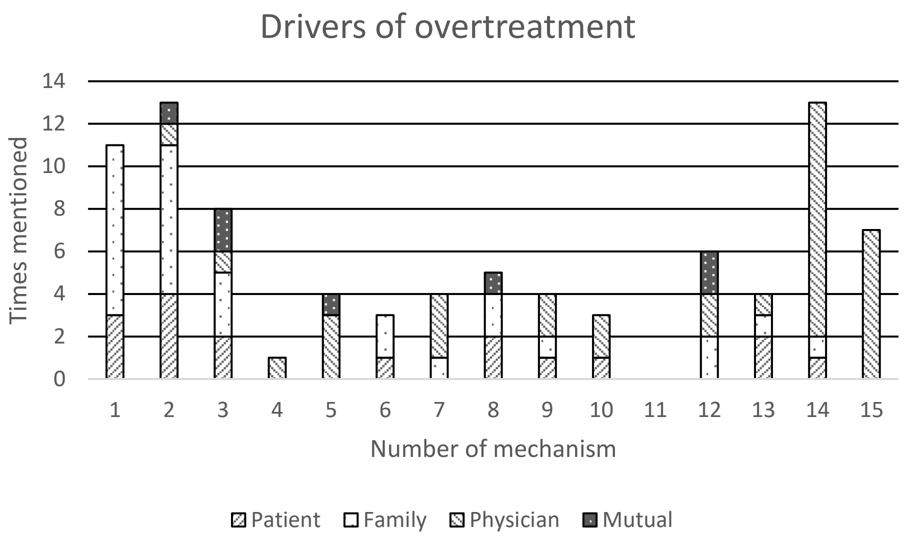

Figure 2. Inhibitors of overtreatment, attributed to different parties

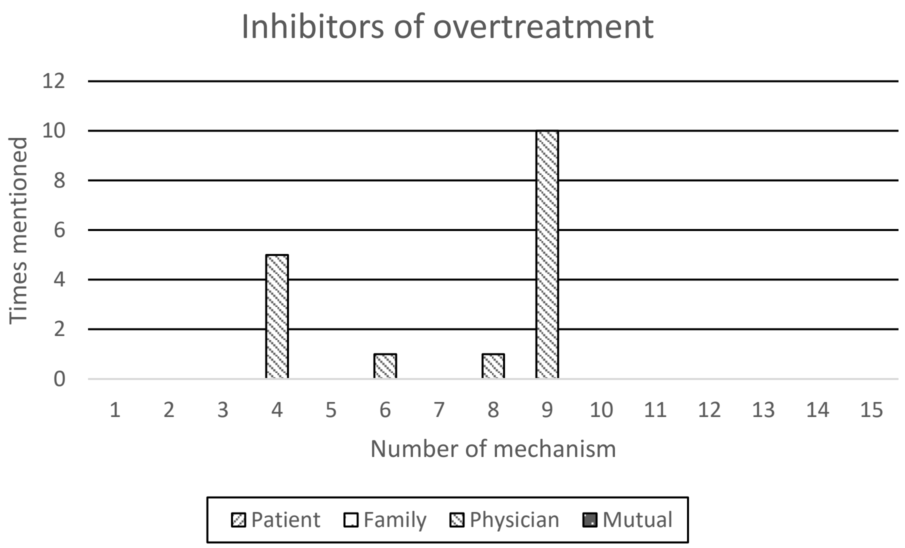

For peer review only

Reporting checklist for qualitative study.

Based on the SRQR guidelines.

Instructions to authors

Complete this checklist by entering the page numbers from your manuscript where readers will find each of the items listed below.

Your article may not currently address all the items on the checklist. Please modify your text to include the missing information. If you are certain that an item does not apply, please write "n/a" and provide a short explanation.

Upload your completed checklist as an extra file when you submit to a journal.

In your methods section, say that you used the SRQRreporting guidelines, and cite them as:

O'Brien BC, Harris IB, Beckman TJ, Reed DA, Cook DA. Standards for reporting qualitative research: a synthesis of recommendations. Acad Med. 2014;89(9):1245-1251.

		Reporting Item	Page Number
**Title**			
	#1	Concise description of the nature and topic of the study identifying the study as qualitative or indicating the approach (e.g. ethnography, grounded theory) or data collection methods (e.g. interview, focus group) is recommended	1
**Abstract**			
	#2	Summary of the key elements of the study using the abstract format of the intended publication; typically includes background, purpose, methods, results and conclusions	2-3
**Introduction**			
Problem formulation	#3	Description and signifcance of the problem / phenomenon studied: review of relevant theory and empirical work; problem statement	6-7
Purpose or research question	#4	Purpose of the study and specific objectives or questions	7

For peer review only

Methods

Qualitative approach and research paradigm	#5	Qualitative approach (e.g. ethnography, grounded theory, case study, phenomenolgy, narrative research) and guiding theory if appropriate; identifying the research paradigm (e.g. postpositivist, constructivist / interpretivist) is also recommended; rationale. The rationale should briefly discuss the justification for choosing that theory, approach, method or technique rather than other options available; the assumptions and limitations implicit in those choices and how those choices influence study conclusions and transferability. As appropriate the rationale for several items might be discussed together.	8
Researcher characteristics and reflexivity	#6	Researchers' characteristics that may influence the research, including personal attributes, qualifications / experience, relationship with participants, assumptions and / or presuppositions; potential or actual interaction between researchers' characteristics and the research questions, approach, methods, results and / or transferability	8
Context	#7	Setting / site and salient contextual factors; rationale	8
Sampling strategy	#8	How and why research participants, documents, or events were selected; criteria for deciding when no further sampling was necessary (e.g. sampling saturation); rationale	8
Ethical issues pertaining to human subjects	#9	Documentation of approval by an appropriate ethics review board and participant consent, or explanation for lack thereof; other confidentiality and data security issues	5
Data collection methods	#10	Types of data collected; details of data collection procedures including (as appropriate) start and stop dates of data collection and analysis, iterative process, triangulation of sources / methods, and modification of procedures in response to evolving study findings; rationale	8-9
Data collection instruments and technologies	#11	Description of instruments (e.g. interview guides, questionnaires) and devices (e.g. audio recorders) used for data collection; if / how the instruments(s) changed over the course of the study	8-9
Units of study	#12	Number and relevant characteristics of participants, documents, or events included in the study; level of participation (could be	8

For peer review only

		reported in results)	
Data processing	#13	Methods for processing data prior to and during analysis, including transcription, data entry, data management and security, verification of data integrity, data coding, and anonymisation / deidentification of excerpts	8
Data analysis	#14	Process by which inferences, themes, etc. were identified and developed, including the researchers involved in data analysis; usually references a specific paradigm or approach; rationale	9
Techniques to enhance trustworthiness	#15	Techniques to enhance trustworthiness and credibility of data analysis (e.g. member checking, audit trail, triangulation); rationale	9
**Results/findings**			
Syntheses and interpretation	#16	Main findings (e.g. interpretations, inferences, and themes); might include development of a theory or model, or integration with prior research or theory	10-14
Links to empirical data	#17	Evidence (e.g. quotes, field notes, text excerpts, photographs) to substantiate analytic findings	10-14
**Discussion**			
Intergration with prior work, implications, transferability and contribution(s) to the field	#18	Short summary of main findings; explanation of how findings and conclusions connect to, support, elaborate on, or challenge conclusions of earlier scholarship; discussion of scope of application / generalizability; identification of unique contributions(s) to scholarship in a discipline or field	15-17
Limitations	#19	Trustworthiness and limitations of findings	17
**Other**			
Conflicts of interest	#20	Potential sources of influence of perceived influence on study conduct and conclusions; how these were managed	4
Funding	#21	Sources of funding and other support; role of funders in data collection, interpretation and reporting	5

The SRQR checklist is distributed with permission of Wolters Kluwer © 2014 by the Association of American Medical Colleges. This checklist was completed on 15. October 2019 using https://www.goodreports.org/, a tool made by the EQUATOR Network in collaboration with Penelope.ai

For peer review only
